# Stochastic system identification without an a priori chosen kinetic model—exploring feasible cell regulation with piecewise linear functions

**DOI:** 10.1038/s41540-018-0049-0

**Published:** 2018-04-11

**Authors:** Martin Hoffmann, Jörg Galle

**Affiliations:** 1 Division of Personalized Tumor Therapy, Fraunhofer ITEM, BioPark I, Am Biopark 9, 93053 Regensburg, Germany; 2 Department of Data Science and Knowledge Engineering, Maastricht University, Bouillonstraat 8-10, 6211 LH Maastricht, The Netherlands; 3 Interdisciplinary Centre for Bioinformatics, University of Leipzig, Härtelstr. 16-18, 04107 Leipzig, Germany

## Abstract

Kinetic models are at the heart of system identification. A priori chosen rate functions may, however, be unfitting or too restrictive for complex or previously unanticipated regulation. We applied general purpose piecewise linear functions for stochastic system identification in one dimension using published flow cytometry data on *E.coli* and report on identification results for equilibrium state and dynamic time series. In metabolic labelling experiments during yeast osmotic stress response, we find mRNA production and degradation to be strongly co-regulated. In addition, mRNA degradation appears overall uncorrelated with mRNA level. Comparison of different system identification approaches using semi-empirical synthetic data revealed the superiority of single-cell tracking for parameter identification. Generally, we find that even within restrictive error bounds for deviation from experimental data, the number of viable regulation types may be large. Indeed, distinct regulation can lead to similar expression behaviour over time. Our results demonstrate that molecule production and degradation rates may often differ from classical constant, linear or Michaelis–Menten type kinetics.

## Computational Bioscience: Cell-regulation appears more diverse than originally thought

Classical cell-regulation models are often imperfectly fitting or even inconsistent with experimental data suggesting inappropriate model assumptions. Martin Hoffmann from Fraunhofer ITEM Regensburg and Jörg Galle from IZBI Leipzig analysed different protein and gene expression data using general purpose piecewise linear functions for system identification. They assessed data corresponding to various experimental techniques for their potential to determine the parameters of their models. Single-cell recordings of expression values over time were most effective for parameter identification. Generally, different and often non-classical cell-regulation models were consistent with the experimental data, even for restrictive error bounds. The authors used virtual treatment experiments to demonstrate that precise knowledge of cell regulation is important for assessing therapy effects. Their findings clearly argue in favour of system identification being performed without an a priori chosen kinetic model.

## Introduction

System identification faces three major indeterminacy issues: (i) true functional equivalence of different regulatory mechanisms; (ii) apparent functional equivalence; and (iii) relative parameter insensitivity conferred by mathematical functions. True functional equivalence is generally limited to an operational range, where any representative is equally practical. In contrast, apparent equivalence is linked to actual measurement data. For example, identifiability depends on the kind of data analysed. As an illustration, basic molecule birth and death processes^1^ result in Poissonian steady state probability distributions for the number of expressed molecules. These distributions are characterised by a single parameter: the mean expression *ν* = *α*/*λ* defined by the production rate *α* and the molecular degradation constant *λ*. Thus, identical steady state distributions can be observed for high and low levels of molecule production and degradation. Nevertheless, the distinction between high and low rates can be relevant in practice, e.g., under limiting cell culture conditions, and requires direct rate measurement. From frequency distributions of expression values, parameters influencing scarcely populated regions are hardly identifiable. Though the same parameters can be determined using single-cell tracking data if the absolute number of observed molecule production and degradation events is sufficiently high.

Choosing the right rate functions can compensate for incomplete data if the underlying regulation type, i.e., the qualitative form of production and degradation rates, is known. Ad hoc selection of specific mathematical functions may otherwise exclude equivalent or even more appropriate regulation. Parametric functions generally show different sensitivities for their individual parameters in different domains. In this respect, piecewise linear functions are advantageous because their *y*-set points influence function values equally if their corresponding *x*-set points are equidistant and non-boundary. Notably, piecewise linear functions can approximate any continuous function, including classical rate functions. In the following, we explore further advantages of this approach using published data from different experimental paradigms.

We start by modelling two experiments by Kashiwagi et al.^2^ on the functioning of a synthetic gene regulatory toggle switch in *E.coli*. The first experiment shows that the switch is effective only if proliferation in cell culture is slowed down by an antibiotic. Otherwise molecular dilution during cell division is dominant and results in an almost homogeneous population of cells that maintain only a few molecules. The second experiment in ref. ^2^ shows that if the vital enzymes GLS-H or DHFR are depleted from the culturing medium, *E.coli* is able to activate exactly that branch of the switch that compensatory produces the required enzyme. We hypothesised that the engineered bacteria might explore expression choices of the implanted plasmid by randomly activating either branch of the switch using a mechanism similar to what we introduced as noise-driven dynamics before.^3–5^ In our simulations, we aimed at validating this hypothesis and employed general purpose piecewise linear functions^6^ to minimise bias through a priori rate function choice. This approach differs from previous work that featured constant, Michaelis–Menten or Hill type rates^7–10^ or introduced step functions comprising only few discrete states.^11^

Employing a second data set of Miller et al.^12^, we particularly focused on the regulation of molecule degradation. To the best of our knowledge, molecule degradation has so far invariably been modelled by a linear term, i.e., assuming the molecular degradation coefficient *λ* to be constant, in system identification studies^7–11,13–15^ despite the fact that actively regulated mRNA and protein degradation, i.e., variable *λ*, has frequently been reported in the literature.^9,16–34^ Thus, we use the data by Miller et al. for a critical evaluation of the linear degradation term assumption.

In both applications, the range of feasible rate functions able to generate expression values similar to the experimental data was rather broad. We thus explored further data types and parameter identification strategies for their power to narrow down the feasible parameter range and thus determine model parameters as uniquely as possible. Direct frequentist evaluation of single-cell tracking data performed best in this respect.

Our major finding is that a large variety of non-standard regulation types are consistent with the experimental data. In particular, production and degradation rates can differ from the classical constant, linear or Michaelis–Menten type. In order to access these additional options for cell regulation, we propose to begin systems identification by applying non-limiting identification approaches in general. Recent and comprehensive reviews on system identification related to this work can be found in refs.^13–15^

In the following, we model the first and second experiments of Kashiwagi et al. (fluorescence cytometry data) on *E.coli* and reanalyse the metabolic labelling data of Miller et al. on *S. cerevisiae* (yeast osmotic stress response). The overt rate indeterminacy issues encountered during these analyses are followed-up by the ensuing comparison of different system identification approaches. Finally, selected virtual treatment experiments demonstrate precise model identification to be required for quantitatively assessing treatment effects in practice. A workflow diagram is provided as Supplementary Figure 2. Individual steps are detailed in Supplementary Methods 4.

## Results

**Fig. 1:**
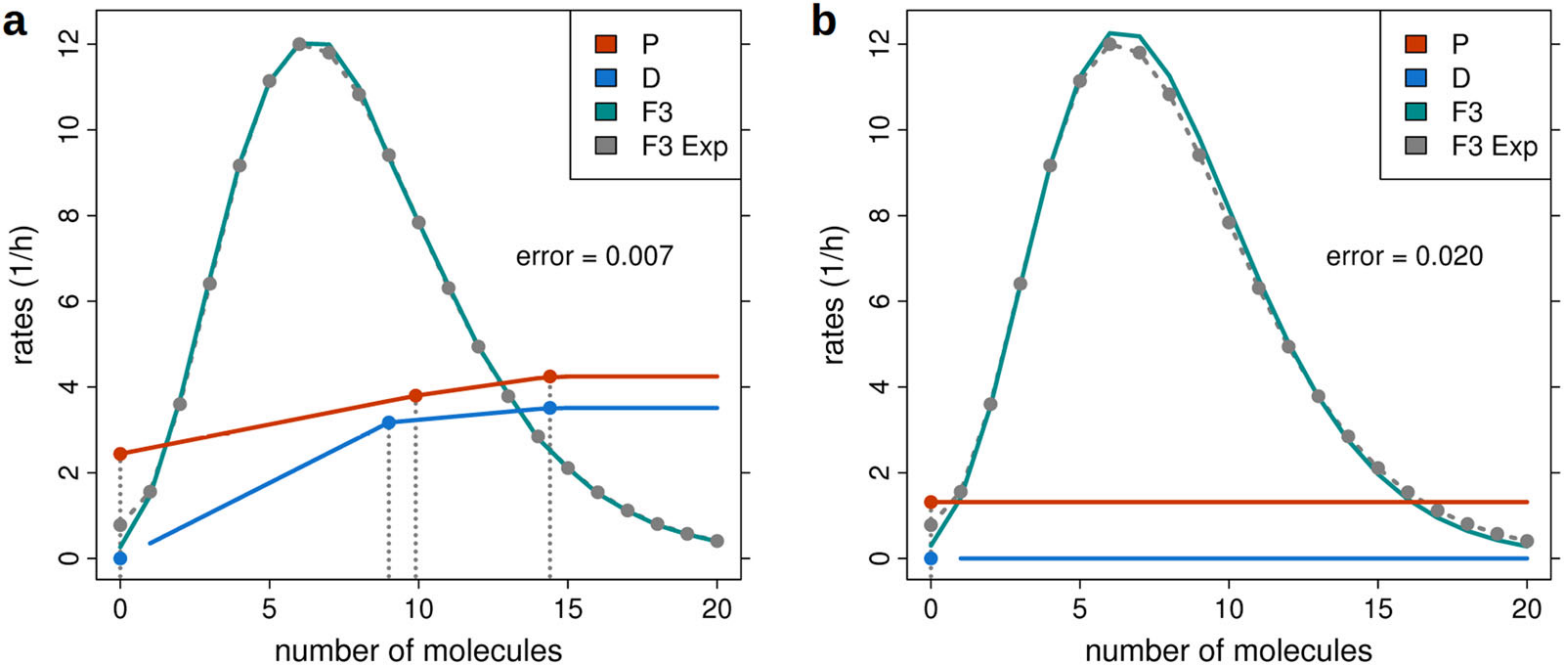
Model properties and data fit. Production (*P)* and degradation (*D*) rates and the simulated (*F*3) and experimental (*F*3 *Exp*) frequency distributions after 3 days of cell culture for minimum error parameters and a maximum number of molecules *N* = 30. **a** Piecewise linear model. **b** Basic birth and death process model (constant *P*, linear *D*). *D* is identified to be zero, which for bacteria has also been assumed in the literature.^38,46^ This contrasts the co-upregulation of *P* and *D* identified in **a**. Thus, very small differences in data fit and varying model assumptions can result in largely different final parameters and lead to opposing conclusions. This is clearly indicative of serious identifiability problems. In **a**, the three individual *x*- and *y*-set points for *P* and *D* are indicated by vertical lines and red and blue points, respectively. Rates are set constant right of the 3rd set-point. For degradation rates the lower boundary (*D*(0) = 0) is omitted from the line graph. Frequencies were scaled to match the upper rate limit (12/*h*). The number of molecules per cell *n* is displayed up to the 99% percentile of the experimental frequency distribution (*n* ≤ 20). Reduced growth rate: 0.65·0.5·ln(2)/h. Original data by courtesy of Kashiwagi and Yomo

We modelled production (*P*), degradation (*D*) and growth (*G*) rates by piecewise linear functions parameterised by respectively three x- and y-set-points (Fig. 1). This allowed to model rate functions with a maximum or minimum, the latter being a key characteristic of noise-driven dynamics.^3^ *P* and *D* were limited to a maximum of 12/h^12,35–37^ while the minimum cell doubling time was set to 2 h matching the growth data of the second experiment of Kashiwagi et al.^2^ and corresponding to a mean growth rate of the modified bacteria of 0.5·ln(2)/h during overnight culture. We denote by *A=P−D* the deterministic and by *B=P+D* the noise term of the dynamics (see Methods for details).

### Non-homoeostatic regulation can promote noise-driven dynamics

**Fig. 2:**
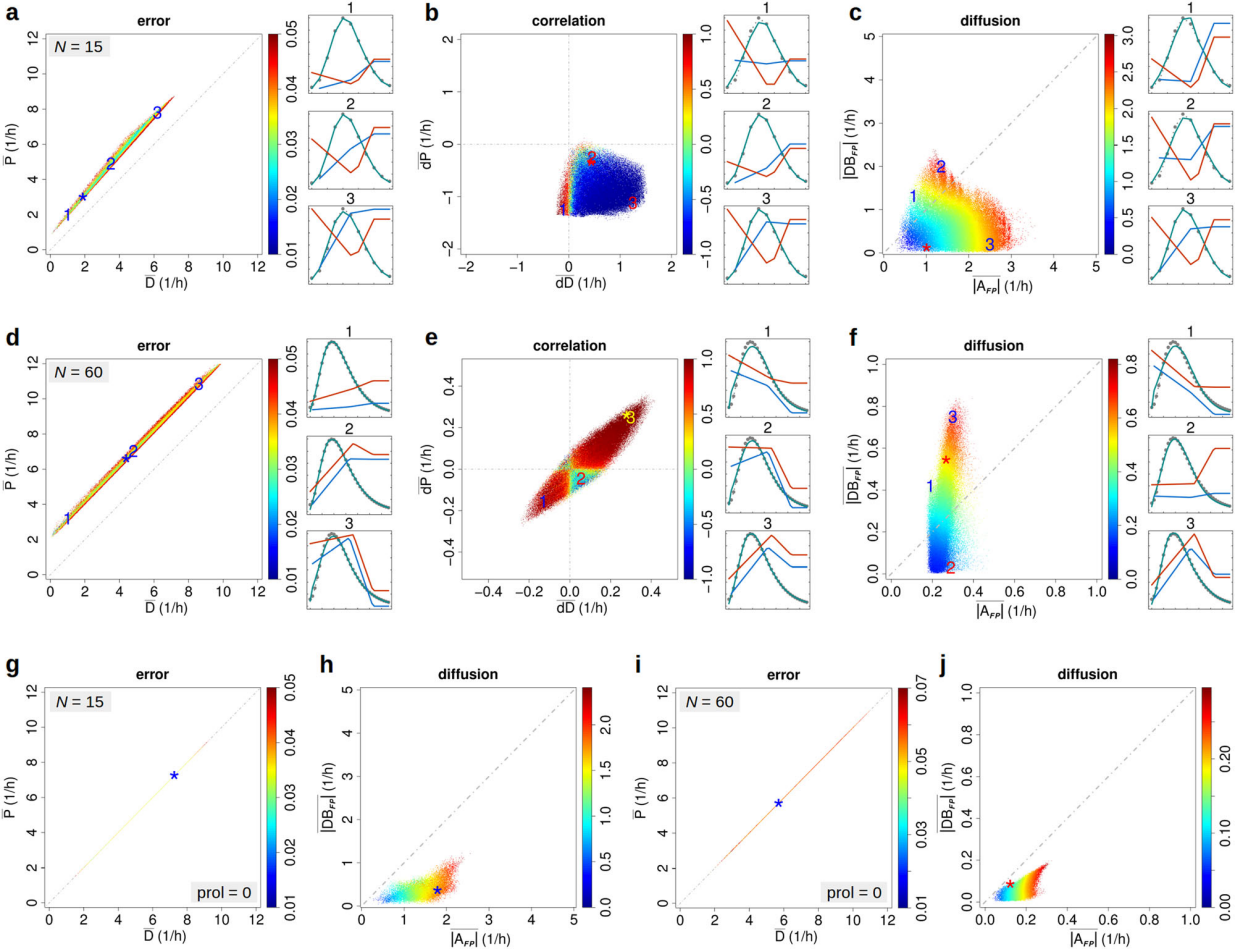
Feasible region (error ≤ 0.05) identified using equilibrium fluorescence cytometry data and depicted according to three different characteristic plots. **a** Data fitting error (colour-coded) of the feasible region as a function of population-averaged production ($\bar P$) and degradation ($\bar D$) rates for *N* = 15. Numerals indicate areas for which representative results are shown in the accordingly numbered small images (display analogous to Fig. 1). The asterisk indicates the error minimum. **b** Population-averaged correlation as a function of the population-averaged slopes of production ($\overline {{\mathrm d}P}$) and degradation ($\overline {{\mathrm d}D}$) rates. **c** Average absolute Fokker–Planck-associated diffusion term as a function of the corresponding average absolute deterministic term $\overline {|A_{{\mathrm {FP}}}|}$ and regulated noise term $\overline {|DB_{\mathrm {FP}}|}$. **d**–**f** Analogous results for *N* = 60. **g**–**j** Results for the same data but assuming zero proliferation during modelling. Panels **g**, **h** relate to **a**, **c**, panels **i**, **j** to **d**, **f**. Colour in 2D plots corresponds to averages according to a 600 × 600 grid. Reduced growth rate 0.65·0.5·ln(2)/h (experimental measurement) in **a**–**f** and assumed zero growth in **g**–**j**. Population-averaged correlation is calculated first per cell cycle phase *i* weighting *P*(*n*, *i*) and *D*(*n*, *i*) by the probability *p*(*n*, *i*) and, second, by averaging across *i* (see Methods). Note that we increased the error limit to 0.07 for *N* = 60 in **i**, **j** because of reduced efficiency of the zero proliferation model. Original data by courtesy of A. Kashiwagi and T. Yomo

We used the first experiment of Kashiwagi et al.^2^ to test whether these data provide any evidence for noise-driven regulation in the system (Methods). The experiment recorded the expression of two reporter genes—each integrated into one arm of a synthetic toggle switch—before and 3 days after growth rate reduction by the antibiotic nalidixic acid. Both recordings represent equilibrium states and were pre-processed as described in Supplementary Figure 3. *y*-set points of production and degradation rates (six parameters) were randomly initialised and optimised during a fixed number of 200 iterations. Random initialisation was repeated until 15,000 trajectories had reached the feasible region defined by an error ≤0.05 (Methods). This corresponded to 100 and 81% of initiating trajectories for the models assuming a maximum number of molecules (as mapped to the maximum fluorescence intensity; see Methods)* N* = 15 and *N* = 60, respectively. Feasible parameter regions were found to be extensive, as evidenced by the wide range of corresponding average production and degradation rates (Fig. 2a, d). Average excess of production over degradation (distance to diagonal) appeared almost constant for all feasible parameters and reflects net molecule production during cell proliferation counteracting molecular dilution, which can on average be assumed to be proportional to growth.^38^

Population-averaged slopes of production and degradation rates (Fig. 2b,e) occupy different parameter regions for *N* = 15 and 60, with a tendency towards co-regulation (similar slopes) at higher *N*. The different (positive/negative) slope combinations are consistent with the population-weighted correlation between rates (Supplementary Methods 5). Figure 2c,f displays the average absolute deterministic, regulated noise and diffusion terms of the Fokker–Planck (FP) equation (Methods). With larger *N*, the feasible region becomes more vertically extended along the *y*-axis indicating a higher probability for noise regulation-dominated dynamics (Methods). Results for *N* = 30 are displayed in Supplementary Figure 4a–c. Corresponding graphs showing colour-coded errors instead of correlation and diffusion are presented in Supplementary Figure 5.

The above results refer to slow proliferation (65% of maximum growth rate), during which the toggle switch was effective (Supplementary Figure 3). Results for fast proliferation, for which no switching was observed due to molecular dilution, were generally similar. However, rate derivatives were much more scattered suggesting that parameter identification was less reliable (likely due to the peaked frequency distribution). Importantly, it was practically impossible (minimum error = 0.25) to simultaneously fit the distributions for fast and slow proliferation by the same production and degradation rates, i.e., when only the observed 30–40% reduction in growth rate^2^ was accounted for. This incompatibility may be due to growth rate-dependent regulation,^39–42^ in particular plasmid-based protein production,^43^ or result from additional side-effects of the DNA replication blocker nalidixic acid, a DNA gyrase and topoisomerase IV inhibitor that affects supercoiling.

Non-proliferating cells do not require net molecule production, which in turn implies a positive deterministic dynamical component (see Methods). We thus expected that noise-driven dynamics—defined by the absence of such deterministic term—would take a larger role in resting cell populations. We tested this hypothesis by assuming zero proliferation in the modelling of the first experiment of Kashiwagi et al.^2^ Counter-intuitively, zero growth completely eliminated the noise regulation-dominated section of the feasible region (Fig. 2h, j, Supplementary Figure 4c and 6c). Also, the required equality of population-averaged production and degradation rates was much more strictly obeyed during zero proliferation (diagonal in Fig. 2g, i) than was their suggested constant difference in proliferating cells (Fig. 2a, d). Notably, randomness introduced by cell division appears negligible in this respect since a molecule number halving model and the binomial distribution in equation (2) gave similar results, as was analogously stated in.^44^

The above results suggest that cell-wise non-homoeostatic regulation, as implied by e.g., transient amplifying states in cell differentiation,^45^ goes along with noise-dominated dynamics. Of course, the data are still consistent with different regulation types.

### Are transient response data actually beneficial for system identification?

Feasible parameter regions as identified from the first experiment of Kashiwagi et al.^2^ are rather large. Since transient response data have been suggested to provide a clear benefit for system identification,^7,46,47^ we tested whether the time series data of the second experiment would shrink the corresponding feasible regions.

**Fig. 3:**
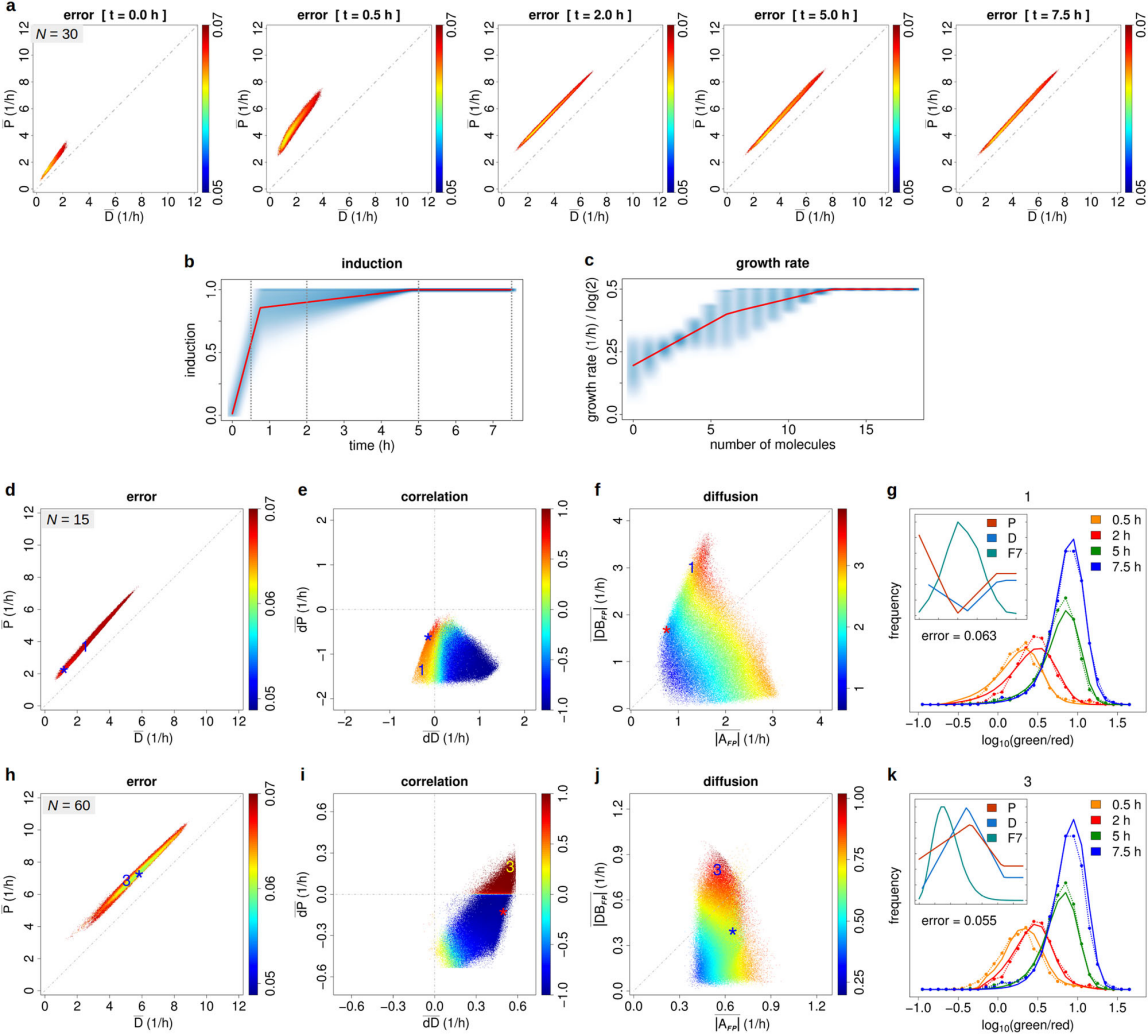
Feasible parameter region (error ≤ 0.07), induction function and growth rate identified using dynamic fluorescence cytometry data. **a** Data fitting error (colour-coded) of the feasible region as a function of population-averaged production ($\bar P$) and degradation ($\bar D$) rates at 0, 0.5, 2, 5 and 7.5 h after medium change (*N* = 30). **b** Induction *I*(*t*) and **c** growth rate *G*(*n*) depicted as smoothed colour-coded density across all feasible parameter vectors (*N* = 30). **d** Fitting error as a function of population-averaged production ($\bar P$) and degradation ($\bar D$) rates for *N* = 15 at 7.5 h. **e** Population-weighted correlation between production and degradation rates as a function of population-averaged slopes of production ($\overline {{\mathrm d}P}$) and degradation ($\overline {{\mathrm d}D}$) rates. **f** Average absolute Fokker–Planck-associated diffusion term (colour-coded) as a function of the corresponding average absolute deterministic term $\overline {|A_{{\mathrm {FP}}}|}$ and regulated noise term $\overline {|DB_{\mathrm {FP}}|}$. **g** Experimental and fitted distributions of green/red fluorescence log-ratios (Supplementary Methods 6) for the four experimental times. The title numeral 1 indicates the corresponding areas in panels **d**–**f**. The inset shows production (*P*) and degradation (*D*) rates, and the simulated distribution of molecules at 7.5 h (*F*7). The error minimum is indicated by an asterisk (*). **h**–**k** Analogous results for *N* = 60. Errors are means across all four time points while correlation and diffusion correspond to 7.5 h. Colours in 2D plots are averages according to a 600×600 grid. Densities in **b**, **c** were calculated by pooling all 2D rate points, e.g., {(*t*_*i*_, *I*_*j*_(*t*_*i*_))} with *i* the discrete time and *j* the parameter index, and calculating kernel densities in 2D using R (blue clouds). Red lines connect mean values per discrete time (**b**) or molecule number (**c**). Original data by courtesy of A. Kashiwagi and T. Yomo

The second experiment of Kashiwagi et al.^2^ provided data on the dynamics of gene induction after medium change (enzyme depletion) in terms of fluorescence intensity log-ratios indicative of compensatory enzyme production (switch activation). Parameter optimisation was performed as before to obtain 15,000 trajectories reaching the feasible region except that individual trajectories were continued for 400 iterations, accounting for the more involved fitting problem, i.e., simultaneous adaptation to four different fluorescence distributions at 0.5, 2, 5 and 7.5 h, and inclusion of 2·2 additional *y*-set points for growth rate and response induction. Modelling an explicitly time-dependent response induction was necessary to account for delayed system response likely due to unmeasured intermediate processes during the transition from fast proliferation (full medium) to reduced proliferation and switch activation (depleted medium). The induction function *I*(*t*) transforms equilibrium rates for fast proliferation (*R*_f_) into those of switch activation (*R*_s_) according to

1 $$R(n,t) = R_{\mathrm f}(n)\,\left[ {\,1 - I(t){\kern 1pt} } \right] + R_{\mathrm s}(n)\,I(t),$$

in which *R* represents *P* or *D*. Proliferation was assumed to be regulated downstream of the vital enzyme glutamine synthetase (GLS-H) and instantly changed over from a constant maximum rate (0.5 ⋅ ln(2)/h; full medium) to a non-decreasing piecewise linear profile *G*(*n*) (depleted medium). The feasible region, now defined by an error ≤0.07 due to the relatively more complex data (ten parameters, four frequency distributions), was reached by 44 and 62% of trajectories for *N* = 15 and *N* = 60, respectively. Figure 3a–c depicts the region dynamics in terms of population-averaged production and degradation rates, response induction and growth rate (*N* = 30). The first two *y*-set points of, respectively, response induction and growth rate were free parameters. The identified curves thus imply that induction is rapid (0.5–1 h) and proliferation at small enzyme numbers is inhibited, consistent with the experimental findings. Figure 3d–f, h–j display results analogous to Fig. 2a–f at time = 7.5 h and Fig. 3g, k illustrates results for the parameter areas indicated by numerals 1 and 3. Both panels correspond to noise regulation-dominated dynamics. In particular, the rates in Fig. 3g have their minima at maximum population density, a key equilibrium characteristic of noise-driven dynamics.^3^ Indeed, this dynamical type should be favoured in the low expression range (here, *N* = 15) because the left boundary condition *D*(0) = 0 implies a natural positive deterministic component (for *P*(0) > 0) required to increase the mean number of molecules. Results for the minimal error state (*) were similar (Supplementary Figure 7a). Corresponding images showing colour-coded errors instead of correlation and diffusion are shown in Supplementary Figure 8.

Details of the experimental procedure and the definition of the feasible region differ slightly between the equilibrated system and the dynamic time series. Nevertheless, in absence of a strictly equal error scale, comparability of results is warranted by the fact that feasible parameters were determined by 15,000 trajectories with mostly comparable success rates in both cases. Despite this minor limitation, our results show that the extent of the corresponding feasible regions in Figs. 2 and 3 is largely comparable.

We stress that the rate degeneracy observed is unlikely to result from model over-parameterisation since already the two parameter classical birth and death model^1^ supports a quasi-unlimited range of rate mean values (Introduction).

### Metabolic labelling data demonstrate strongly regulated degradation

Population-averaging in general implies loss of molecular detail. Nevertheless, observing average system behaviour over time can signify regulatory relationships that are equally valid also at the single-cell level, at least for unimodal, strongly peaked population distributions. We pre-processed the metabolic labelling data of Miller et al.^12^ as described in Supplementary Methods 4 and interpolated the resulting expression and rate time series by smoothing splines. In the following, we drop overbars for $\bar P$, $\bar D$, $\bar A$ and $\bar B$. In addition, we assume that mean cellular gene expression $\bar n = T/M$ is equivalent to the total number of molecules *T* as quantified by normalised microarray data.

**Fig. 4:**
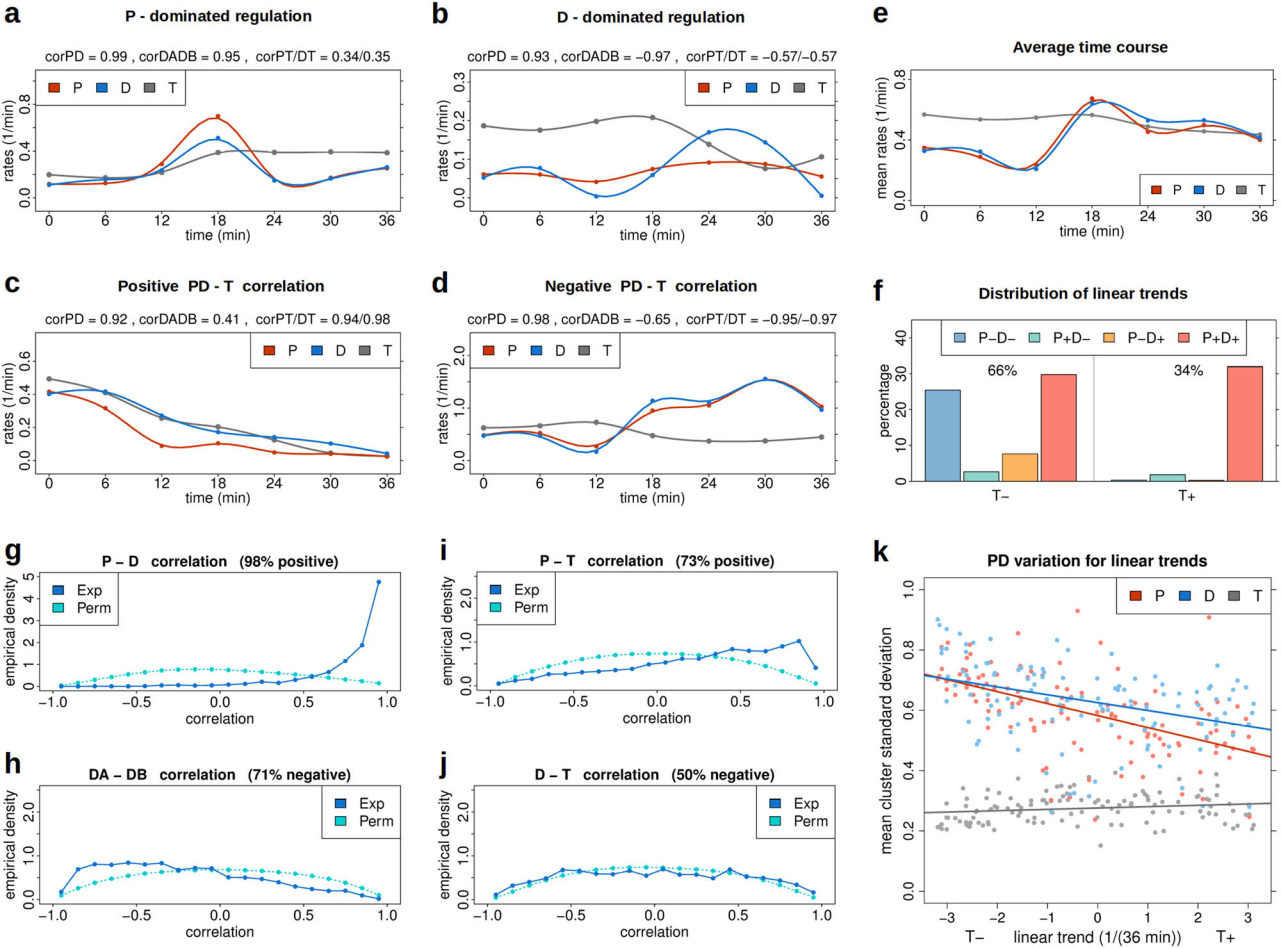
Reanalysis of yeast metabolic labelling data on osmotic stress response. **a**–**d** Illustrating examples for *P*-dominated and *D*-dominated regulation as evidenced by positive (**a**) and negative (**b**) DA-DB correlation (corDADB) and positive (**c**) and negative (**d**) correlation between *P*/*D* and *T* (corPT/DT). The actual correlation values are indicated in the headlines together with the correlation between *P* and *D* (corPD). **e** Mean time course of *P*, *D* and *T* averaged across 2065 genes. **f** Distribution of linear trends (signs of linear regression slopes). Overall decreasing (*T*−) and increasing (*T*+) expression make up 66 and 34% of trajectories, respectively. These are further stratified according to combinations of decreasing and increasing production (*P* ±) and degradation (*D* ±) rates. More than 1/3 of trajectories (37%) are characterised by decreasing *T* and increasing *D*. Parallel *P* and *D* increase (62%) may largely result from the strong upregulation step between minutes 12 and 18 (**e**). **g**–**j** Distribution of Pearson correlation values between *P* and *D* (**g**), time derivatives *DA* and *DB* (**h**), *P* and *T* (**i**) and *D* and *T* (**j**). Shown are distributions for the experimental time ordering (Exp) and corresponding reference distributions generated by enumerating all 5040 time point permutations (Perm). **k** Mean within-cluster standard deviation of *T*, *P* and *D* for 122 time course clusters (points) as a function of the clusterwise linear expression trend (*T*). Also shown are linear regression lines. Clusters were defined by complete linkage clustering of expression trajectories (*T*) according to a maximum correlation distance of 0.15. Time courses were standardised across time (mean=0, standard deviation=1). Within-cluster standard deviations were first calculated for each time point across genes before averaging across time. Curves for *T* in (**a**–**d**) are scaled by a factor 0.1. Original data by courtesy of B. Schwalb and P. Cramer

Figure 4a–d shows example trajectories of production (*P*) and degradation (*D*) rates and gene expression (*T*) as observed for osmotic stress response in *S.cerevisiae*. These data illustrate the presence of production-dominated and degradation-dominated regulation as well as positive and negative correlation between both *P* and *T* and *D* and *T* (see Supplementary Figure 9a–l for additional examples). *P*-dominated and *D*-dominated regulation can be identified by respectively positive and negative correlation between the time derivatives of the terms *A* (*DA*) and *B* (*DB*) (Methods).

Figure 4e depicts the average time course across the 2065 quality-filtered genes showing high overall co-regulation of production and degradation rates. This phenomenon has similarly been reported in a number of recent publications^16–22,31^ and was suggested to implement rapid environmental adaptation.

Linear fits of the individual trajectories indicate that most trajectories (66%) show overall decreasing gene expression over time while being mostly associated with increasing degradation (D+, 37%) (Fig. 4f), clearly contradicting the linear degradation model.

The strong rate co-regulation observed in the sample trajectories is corroborated by the corresponding distribution of gene-wise correlation values (Fig. 4g). Here, a reference distribution based on enumerating all time point permutations was added (Supplementary Methods 4). The distribution of *DA* − *DB* correlation values (Fig. 4h) demonstrates preference for *D*-dominated regulation.

Figure 4i, j show histograms for correlation values between *P* and *T* and *D* and *T*, respectively. *P* is mainly positively correlated with *T*, but the association between *D* and *T* is fully balanced. Once more, this contradicts linear degradation, for which a strong bias towards positive correlation should be observed.

To quantify the diversity of regulation types we clustered the genes into 122 groups of similar expression time courses and calculated mean within-cluster standard deviations (Supplementary Figure 9s, t). Figure 4k displays these standard deviations as a function of the cluster-wise linear expression trends. It demonstrates low variability for *T* and decreasing variation for *P* and *D*. If expression time courses were tightly linked to specific regulation types, within-cluster variation of *P* and *D* should not much exceed that of *T*. However, variation of *P* and *D* is about 3–4-fold higher for negative trends and 2–3-fold higher for positive trends. Especially, variation of *D* at negative trends tends towards unity—the expected value for standard random data.

Taken together, the results of this section clearly demonstrate that for the present data mRNA degradation is regulated in parallel to mRNA production and is overall uncorrelated with mRNA level.

### What should we measure? A comparison of different system identification approaches

The results of the previous sections plainly illustrate that critical evaluation of different data types and evaluation methods assessing their ability to pin down system-specific parameter values is urgently needed. We thus screened six different approaches, five of which involve metabolic labelling or single-cell tracking, and compared method performance based on specifically generated semi-empirical synthetic data (Methods).

For this purpose, the minimum error states (*) in Fig. 3 (Supplementary Figure 7) were used to create synthetic reference data of different types from the same model parameters. Subsequently, we evaluated the investigated methods using all feasible parameters identified in the penultimate section (Fig. 3) and did not perform new method-specific identification runs for these data (Supplementary Methods 4). This procedure has the great advantage of all methods being evaluated for exactly the same parameters, while on the other hand, method-specific feasible regions may be incompletely mapped.

The six identification methods/error types relate to differences regarding molecule frequency distributions in 1D (Freq 1D), as used above, and 2D (Freq 2D), related to metabolic labelling, differences in population-averaged rates (Rates PAV), also relating to metabolic labelling, differences in rates as derived from ideal (Rates SCT id) and simulated (Rates SCT) single-cell tracking (SCT), as well as the negative log-likelihood (LogLike SCT) of the synthetic SCT data (see Methods and Supplementary Methods 4,8–10 for details).

**Fig. 5:**
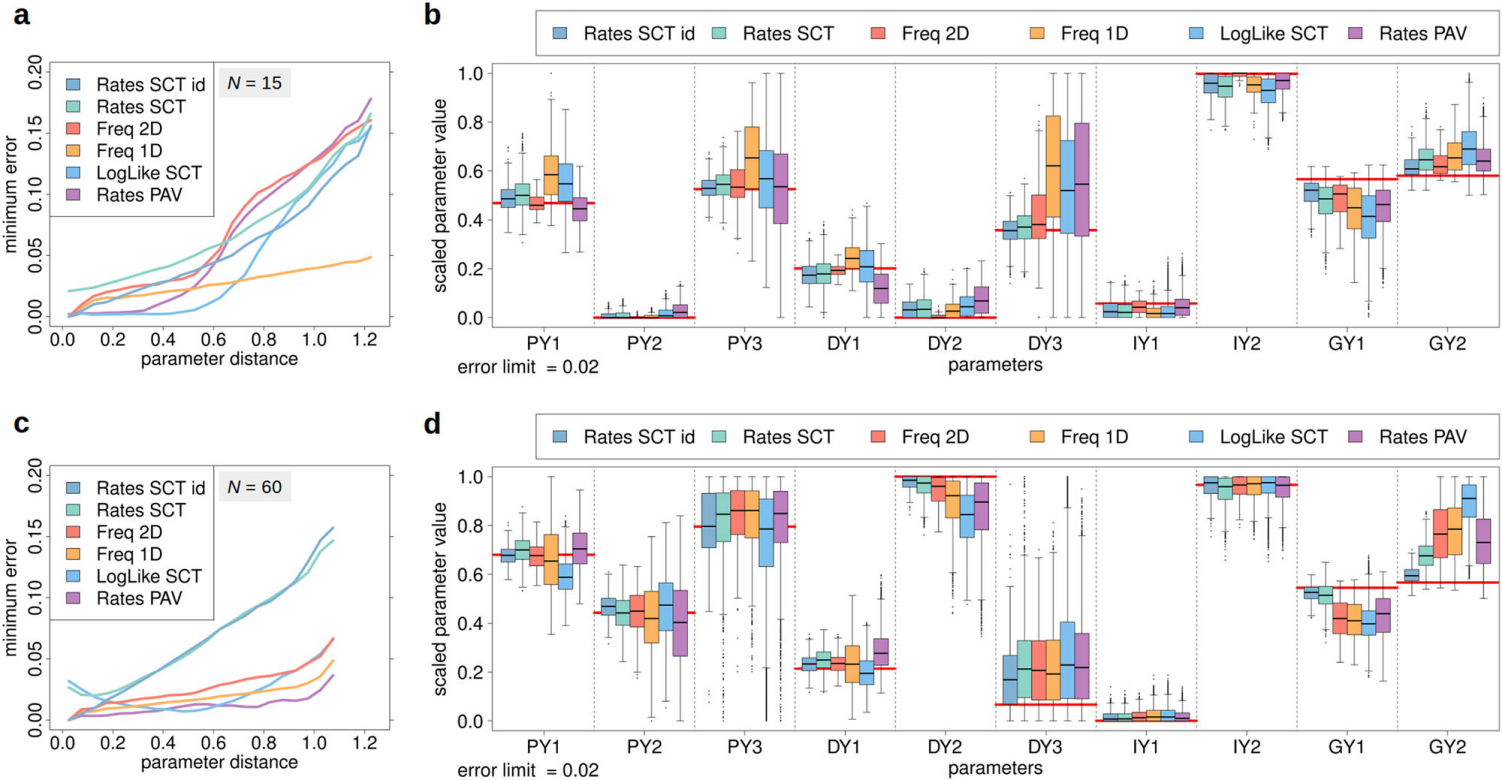
Comparison of parameter identification methods. **a** Minimum error as a function of euclidean distance to the reference parameter vector for six parameter identification approaches: Freq 1D, Freq 2D, Rates PAV, Rates SCT id, Rates SCT and LogLike SCT for *N* = 15. Minimum errors were calculated for different distances by stratifying errors according to centred distance windows (width = 0.05) and averaging across the lower 1% error percentile. Freq 1D and Freq 2D are naturally on the same scale. Rates PAV, Rates SCT id, Rates SCT and LogLike SCT were scaled to Freq 2D. For illustration purposes, the minimum error of Rates SCT was not adjusted to be zero. **b** Boxplot for all parameter sets (0/1-scaled *y*-set points PY1-GY2) corresponding to method-specific errors ≤0.02. Red horizontal lines indicate true parameter values. Generally, the highest parameter variability is observed for the respective 3rd *y*-set points of production (PY3) and degradation (DY3) rates corresponding to regions scarcely populated during the entire time course. Different from panel **a**, Rates SCT is fully scaled to Freq 2D, i.e., all methods have zero minimum error. (**c**, **d**) Analogous results for *N* *=* 60

Figure 5a demonstrates that all investigated methods are subject to identifiability issues. Only very small deviations from the reference data narrow the range of feasible parameters sufficiently down. E.g. for *N* = 15, parameters with a distance to the reference parameter set of up to ~1/3 of the maximum distance show very small minimal errors (<0.05) irrespective of the specific method used. This implies that already small experimental inaccuracies may have a strong effect on identified parameter values.

Figure 5b shows box plots for all parameters corresponding to method-specific errors ≤0.02. Analogous results for *N* = 60 are displayed in panels c and d. The outcome for *N* = 30 is even more obvious (Supplementary Figure 12a,b).

Because of the low identifiability of parameters PY3 and DY3 these were excluded from distance calculations in (a,c). This measure somewhat increases the error-distance slope for methods, for which PY3 and DY3 were variable (b,d). Results based on all parameters are shown in Supplementary Figure 12c-e.

Notably, Rates SCT and LogLike SCT derive from identical data (Supplementary Methods 4). The better performance of Rates SCT can evidently be attributed to the additional stratification (normalisation) step that in effect separates the overall identification task into smaller sub-problems—here, one for each number of molecules (Supplementary Methods 9). This appears especially useful for regions with relatively few events that are otherwise outnumbered in the global likelihood (Supplementary Methods 8).

Supplementary Figure 13 reports on additional results of local Hessian sensitivity analysis, indicating that for Freq 1D and Freq 2D the difference between production and degradation rates is the single most important feature.

The results of this section suggest that Rates SCT id, Rates SCT and Freq 2D are associated with the lowest bias and dispersion and thus appear as the most accurate identification methods. Accordingly, single-cell techniques that have increasingly become available in recent years can actually be expected to become a powerful tool for system identification.

### Virtual treatment experiments

The problems of systems identification described above have practical importance specifically for human biological system intervention. In the following, we illustrate this issue by virtual treatment experiments.

**Fig. 6:**
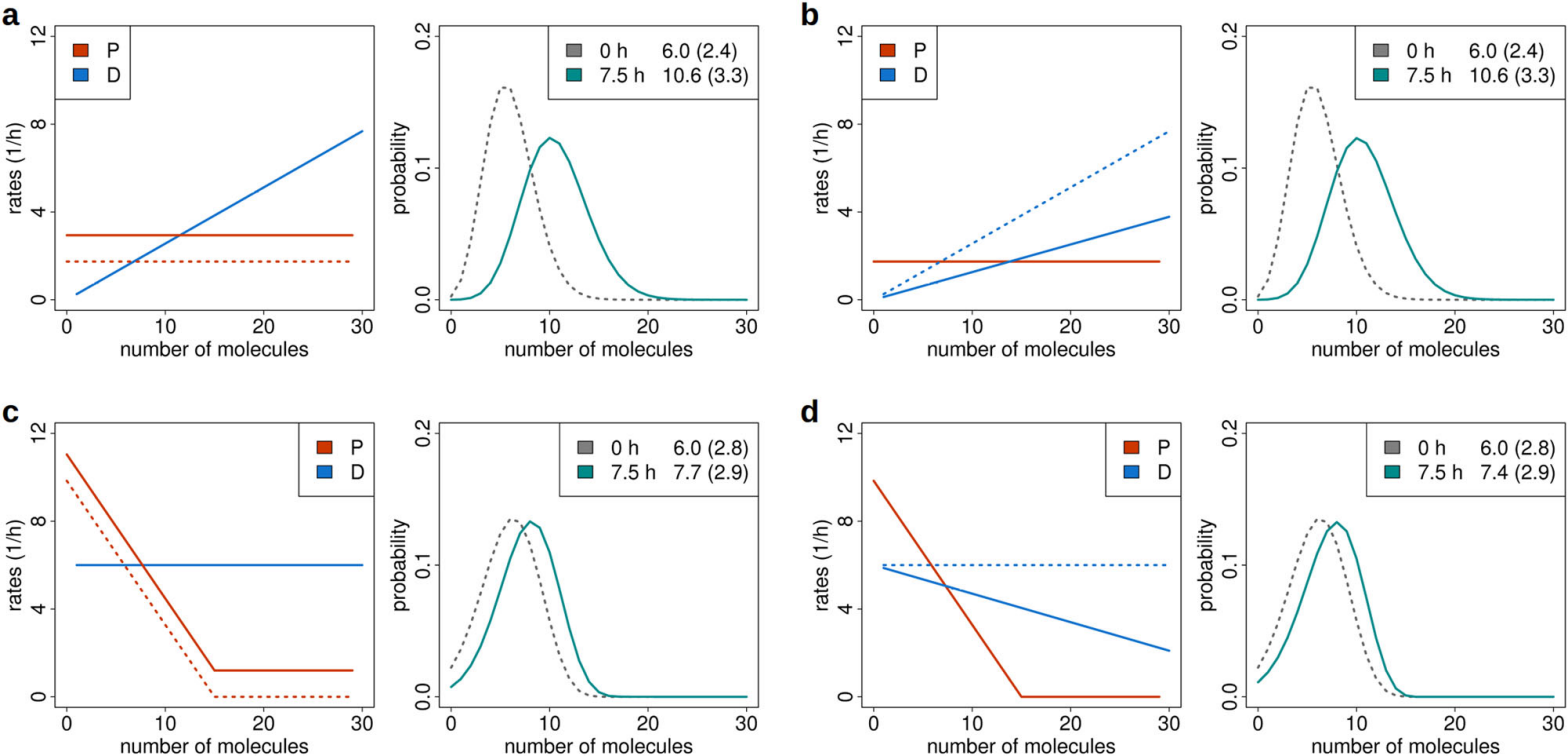
Response of different regulation types to identical treatment. **a** A constant increase in production rate (red dashed line changed to red solid line) is applied to the ground state (time 0 h, mean 6.0, standard deviation 2.4) of a molecule birth and death model, resulting in increased mean (10.6) and standard deviation (3.3) after 7.5 h. **b** The same effect is achieved by a corresponding reduction in the molecular degradation rate (blue dashed line changed to blue solid line). **c**, **d** Identical treatments are applied to a model in which molecule production is downregulated with the number of molecules, while the degradation rate remains constant. These scenarios correspond to much weaker responses: the mean shift by 4.6 in **a**, **b** compares to only 1.7 in **c** and 1.4 in **d**. The standard deviation increases by 0.9 in **a**, **b** but remains quasi unchanged (shift by only 0.1) in **c**, **d**. These results similarly apply for the respective reverse treatments as the system is almost completely equilibrated after 7.5 h. No cell proliferation was assumed

In Fig. 6a the constant production rate *P* = *α* of a classical birth and death process is increased by 63%, e.g., by addition of a transcription factor, chromatin modifier or inhibition blocker. This results in a positive shift regarding the mean number of molecules by 77%. In Fig. 6b the same increase is accomplished by diminishing the molecular degradation constant *λ* by 51%, e.g., by adding capping enzymes or by inhibition of exonucleases, ubiquitin ligases or proteasomes. In panels c and d the same treatments are applied to regulation, in which both* α*(*n*) and *λ*(*n*) = 6/*n* (*n* ≥ 1) are down-regulated upon molecular crowding. The resulting boost in mean is then only about 1/3 of that in (a,b). Moreover, the standard deviation that increases by 38% in (a,b) remains quasi constant in (c,d). Analogous results for two additional regulation types are presented in Supplementary Figure 14.

Thus, treatment response can substantially depend on how precisely regulation is implemented in different cell types or individual patients. This finding clearly argues in favour of system identification being performed without an a priori chosen kinetic model in the first place.

## Discussion

We applied stochastic system identification to the flow cytometry data of Kashiwagi et al.^2^ on a synthetic toggle switch in *E.coli*. Our analysis demonstrated that many different regulation types were consistent with these data, i.e., produced model output showing only minor deviations. This in turn implies that system identification results are sensitive to experimental imprecision of the same order. To assess whether other data types and evaluation methods can amend this situation, we generated diverse semi-empirical synthetic data sets. The corresponding method screen indicated that primarily rate reconstruction from single-cell tracking and, secondary, frequency distributions derived from single-cell metabolic labelling are the most accurate approaches for parameter identification. In contrast, the use of population-averaged rates and likelihoods predicted by single-cell tracking data performed less favourably.

The above results were obtained by application of quasi unconstrained piecewise-linear rate functions that generally differ from commonly employed expression models such as classical birth and death processes or Michaelis–Menten type kinetics. In particular, degradation rates are still widely assumed to be linear. In contrast, our reanalysis of the metabolic labelling data of Miller et al.^12^ during yeast osmotic shock response clearly demonstrated that degradation rates were tightly coupled to production rates, as likewise reported in the literature,^16–22,31^ and can be regulated at least equally strongly. Correlation between trajectories of degradation rate and gene expression showed a quasi-random, sign-balanced distribution across 2065 genes, while wholly positive correlation would be expected if degradation was indeed linear (i.e., unregulated).

A number of factors can influence molecule degradation, ranging from priming by adaptor proteins,^23,24^ cooperative or stepwise degradation,^1,9,33^ storage in granules and P-bodies,^25,31^ effects of regulatory RNAs and RNA-binding proteins^26,30,31^ to overall resource limitation.^27–29,34^ It has repeatedly been pointed out that mRNA and protein degradation are at least equally import for cell regulation as are transcription and translation.^31,32,34^ Thus, general application of linear degradation in biological system identification appears an oversimplification that can result in substantial identification bias. Notably, regulated degradation can also impact the assessment of transcriptional and translational noise.^7–9^

Employing more versatile identification approaches like the one presented in this study explores wider regulatory possibilities that more likely include the true regulation type as long as data overfitting can be excluded. Here, we counteracted overfitting of noisy data by smoothing the fluorescence intensity profiles and limited model over-parameterisation by considering only two or three parameters per rate. For production and degradation rates, a minimum of three parameters was necessary to test whether non-monotonous rate functions, specifically those with a minimum (related to noise-driven dynamics), could be consistent with experiment. Occasionally, we observed pronounced rate changes in sparsely populated regions at large molecule numbers. This might be indicative of low parameter identifiability and could, if desired, be amended by implementing smooth function variation depending on data density.

We do not oppose the common preference for the simplest model. However, it cannot be taken as axiomatic that Occam’s razor selects the true solution. Our reanalysis of the metabolic labelling data of Miller et al. provides clear evidence for regulated mRNA degradation. Evidently, system identification can best be tackled by appropriate measurement and evaluation techniques, like single-cell tracking (reaction event monitoring), that allow precise parameter identification of basic models.

We propose that rate functions successfully identified by rather flexible identification approaches can subsequently be represented by more specialised mappings to reduce the number of parameters as needed. This two-step procedure largely decouples the problems of true and apparent regulatory equivalence from those of relative parameter insensitivity as conferred by mathematical functions (Introduction). Nevertheless, the number of effectively unconstrained (independent) parameters needs to be kept as low as possible to enable quasi exhaustive parameter searches, for which we provided an expert monitoring method.

The starting point and a basic motivation of this study was to further explore our previous hypothesis that noise regulation can be a major driving force of cell dynamics.^3–5^ The noise regulation model assumes that the deterministic part of the dynamics is negligible while the main effects result from local variations in noise level, rendering low noise states population attractors. This idea was later also termed noise-controlled cell regulation by Pujadas and Feinberg.^48^ A main benefit of such design is that regulation need not be learned and hard-wired beforehand but can be explored in situ through immediate feedback monitoring. This principle was similarly implemented in our novel noise-driven optimisation (NDO) method showing very good performance.

To capture noise regulation on top of deterministic growth processes in highly proliferative cells like *E.coli*, we supplemented our original definition of noise-driven dynamics by the more general term noise-dominated dynamics as quantified by corresponding terms of the Fokker–Planck equation. Surprisingly, we found indications of the possibility that noise-dominated regulation may indeed be favoured in proliferating cell populations. Corresponding changes in the noise content of cellular regulation could also be implied in other processes involving variation of proliferation intensity, like batch or fed-batch cell culture, eukaryotic cell differentiation^45^ or cancer.

The general need to identify production and degradation rates is illustrated by our virtual treatment experiments. Evidently, the influence of different regulation types will be more prominent in real-world multi-dimensional settings requiring considerable future efforts to develop clear-cut and effective experimental and mathematical methods for biological systems identification.

## Methods

### General equations

We aimed at modelling fluorescent protein frequency distributions *F* matching experimental cytometry data listing cell counts per binned fluorescent intensity value. For this purpose, we employed a rate equation that accounts for cell proliferation using the multi-phase cell cycle model of Leon et al.^49^ For the number of molecules *n* = 1...*N* per cell and cell cycle phases *i* = 1…*k*, these equations read

2 $$\begin{array}{ccccc}\\ \frac{{{\mathrm d}F(n,i)}}{{{\mathrm d}t}} = & \left[ {\,E_n^{ - 1} - 1\,} \right]\,P(n,i)\,F(n,i) + \left[ {\,E_n^{ + 1} - 1\,} \right]\,D(n,i)\,F(n,i) \hfill \\ \\ & \kern-9pt- \,G(n,i)\,F(n,i) + \left\{ {\begin{array}{*{20}{c}} {\,2\,\mathop {\sum}\limits_{m = n}^N {G(m,k)\,b(n|m,\,1/2)\,F(m,k)} \quad for\,i = 1} \\ {G(n,i - 1)\,F(n,i - 1)\quad \quad \quad for\,i = 2,\,...,\,k} \end{array},} \right.\\ \end{array}$$

with $R(n,i) = R\left( {\left\lfloor {n/v_i} \right\rfloor } \right)$ for *R* = *P*,*D*,*G* indicating the production (*P*), degradation (*D*) and growth (*G*) rates, *v*_*i*_ = 1 + (*i* − 1)/(*k* − 1) defining the cell volume, and $\left\lfloor {n/v_i} \right\rfloor$ the lower integer concentration. $E_n^s\,f(n,i) = f(n + s,i)$ denotes the discrete *n* shift operator and *b*(*n*|*m*,1/2) the symmetric binomial distribution accounting for molecule partitioning among daughter cells.^35,36^ Cell cycle phases allow the modelling of varying cell volume during cell proliferation. According to previous findings,^3^ *k* = 5 is used throughout. For comparison with experiment, *F* is marginalised across cell cycle phases, i.e., we use *F*(*n*) = ∑_*i*_*F*(*n*,*i*). Notably, equation (2) is similar to a chemical master equation (CME).^50^ However, *F* is a frequency distribution summing up to the (time-varying) total number of cells in the system instead of unity. Hence, we use the term rate equation for clarity. Indeed, equation (2) can be derived from an extended CME also accounting for the number of cell divisions (Supplementary Methods 1). In this context, it describes the dynamics of the mean number of cells that harbour *n* molecules and proceed in cell cycle phase *i*, irrespective of cell division history. For zero growth, the number of cells is constant and equation (2) becomes equivalent to a one-dimensional CME for the probability distribution *p*(*n*). A second order approximation to the CME is the Fokker-Planck (FP) equation (Ito form) that, using the deterministic term *A*(*x*) = *P*(*x*) − *D*(*x*) and the noise term *B*(*x*) = *P*(*x*) + *D*(*x*), can be written as (Supplementary Methods 1)

3 $$\frac{{{\mathrm d}p(x)}}{{{\mathrm d}t}} = \begin{array}{*{20}{c}} {\left[ {\frac{\partial }{{\partial x}}\left( { - A(x)p(x)} \right)} \right]} & { + \left[ {\frac{\partial }{{\partial x}}\left( {\frac{{\partial B(x)}}{{\partial x}}p(x)} \right) - \frac{1}{2}\frac{{\partial ^2B(x)}}{{\partial x^2}}p(x)} \right]} & { + \frac{{B(x)}}{2}\frac{{\partial ^2p(x)}}{{\partial x^2}},} \\ {deterministic} & {regulated\,noise} & {diffusion} \end{array}$$

in which we denoted the first summand as FP-associated deterministic term (*A*_FP_(*x*)), the second as regulated noise term (*DB*_FP_(*x*)) and the third as diffusion term. Equation (2) is solved by Euler forward integration (time step 1 min). The FP-terms in (3) are then evaluated using the results of (2).

### Types of dynamics

We link the regulated noise term *DB*_FP_(*x*) with noise-driven dynamics^3^ and generally excluded boundaries in actual function evaluations. In equation (3), −*A*(*x*) and ∂*B*(*x*)/∂*x* take similar roles. Nevertheless, according to the time derivative of the total number of molecules *T* (Supplementary Methods 7)

4 $$\frac{{{\mathrm d}T}}{{{\mathrm d}t}} = M\left( {\bar P - \bar D} \right) = M\bar A,$$

a non-zero population-averaged deterministic term $\bar A$ is required to change *T*. Indeed, if $\bar A$ vanishes the average number of molecules per cell $\bar n = T/M$, with *M* the number of cells, will dillute out in proliferating populations. Without loss of generality, *A*(*x*), *B*(*x*) and the FP-terms in (3) can also be applied to growing cell populations and multiple cell cycle phases. However, we used these terms to exclusively characterise the cell regulation part of molecule expression (rates *P* and *D*) since (i) we did not explicitly account for the distribution of the number of cell divisions (associated with growth rate *G*), for which also no measurement data were available, and (ii) the non-local jump processes during cell division (basically halving of molecule numbers) are not amenable to the FP approximation.^50^ Yet, our conclusions on cell regulation remain unaffected by this choice as long as molecule repartitioning among daughter cells can be considered an unregulated random process^35,36^ or effects of asymmetric cell division average out as per population or over time. We use the population-averaged rates $\bar P$ and $\bar D$, their derivatives $\overline {{\mathrm {d}}P} = \overline {\partial P/\partial x}$ and $\overline {{\mathrm {d}}D} = \overline {\partial D/\partial x}$, and the average absolute deterministic term $\overline {|A_{{\mathrm {FP}}}|}$ and regulated noise term $\overline {|DB_{{\mathrm {FP}}}|}$ to characterise the feasible parameter region by three different 2D graphical plots. Compared to direct parameter display, this has the advantage of immediate meaning and relative independence of mathematical representation. Additionally, we introduce the term noise regulation-dominated dynamics for regulation in which $\overline {|DB_{{\mathrm {FP}}}|} > \overline {|A_{{\mathrm {FP}}}|}$, while noise-driven dynamics^3^ is defined by *A*(*x*) = 0, i.e., equality of *P*(*x*) and *D*(*x*) as a function.

### Parameter identification

Scaled parameters (range [0,1]) were randomly initialised and optimised by minimising the sum of absolute deviations between simulated and experimental frequency distributions. The final scaled error was obtained by dividing by two times the sum of the experimental frequencies, which is a strict error bound for relative frequencies. *x*-set points were adapted in preliminary runs and later fixed. Random initialisation for *y*-set points was repeated until a certain number of trajectories had reached the feasible region defined by an upper error limit.

In view of experimental inaccuracy^51^ (Supplementary Figure 3a), global mapping of the feasible region appeared most appropriate. Accordingly, local sensitivity analysis relative to single best fitting parameters is provided as supplementary information. We discretised the parameter space into a grid of 16 bins per dimension and compiled a list of feasible region bins into which minimal error parameters were recorded.

To reduce overfitting of noisy data, we smoothed the experimental fluorescence intensity histograms by analytical functions (Supplementary Figure 3b, Supplementary Methods 4). Nevertheless, experimental bias cannot generally be excluded. Some spillover between fluorescence channels is clearly present in the data (Supplementary Figure 3a). For matching experimental and simulation results, fluorescence intensities were mapped to molecule numbers using an affine function (Supplementary Methods 2). We investigated three different molecular ranges defined by the maximum number of molecules per cell *N*. Specifically, we designated the sequence *N* = 15,30,60 for analysis and placed the distributional mass within the lower 2/3 of the molecular range to exclude upper boundary effects. This design corresponds to the lower range of the 10–1000 signalling proteins per cell reported for *E.coli*,^52^ which is both most relevant for stochastic process modelling (Supplementary Figure 9u) and computationally efficient.

### Noise-driven optimisation (NDO)

In order to compromise between parameter identification and parameter space exploration (scouting)^53^ we developed a new optimisation method termed NDO. Basically, NDO generates new exploratory parameters by perturbing previously encountered lowest error reference parameters according to a normal distribution whose variance decreases with decreasing reference error (Supplementary Methods 3). Performance of NDO was best compared to the Simplex (Nelder–Mead) algorithm and explored markedly more states than the BFGS (Broyden–Fletcher–Goldfarb–Shanno) approach. Conjugate Gradient and Simulated Annealing were clearly outperformed (Supplementary Figure 1). By assessing detected feasible region size as a function of the number of random initialisations, we also demonstrated that our parameter search was almost exhaustive up to intermediate levels of parameter space fine graining (Supplementary Figure 1).

### Modelling the toggle switch and molecule degradation

Similar to Kashiwagi et al.^2^, we assume instant inhibition of the contra-lateral arm of the toggle switch after medium change. In the case considered here, the glutamine-depleted medium favours the GLS-H branch of the toggle switch, which in turn shuts down DHFR. This implies immediate reduction to a single species system that exclusively describes the induction of the compensatory enzyme (here GLS-H). This view is supported by results of Tsuru et al.^54^ demonstrating analogous adaptation of *E.coli* based on a single gene construct. Our model is designed to describe protein auto-regulation thus treating intermediate processes like the corresponding mRNA dynamics implicitly. This can partly be justified by the fact that transcription and translation occur simultaneously in bacteria. Yet, our ansatz remains a proxy for gene activation by intermediate processes since the plasmid-encoded metabolic enzymes themselves cannot induce their own *tetA* and *trc* promoters. Furthermore, we assume fast promoter switching dynamics,^55^ thus neglecting promoter state-dependent expressional bursts, that nevertheless, appear to predominate in mammalian cells.^56^ Multiple promoter or RNA polymerase activation states, as e.g., mediated by sigma-factor dynamics in bacteria,^57^ were recently reported to have a burst-balancing effect.^56^ Nevertheless, we cannot presently exclude that promoter switching^35^ may have an influence on system identification results. Also, due to the experimentally chosen co-expression construct^2^ degradation rates refer to the fluorescent reporter proteins instead of the target enzymes. However, we expect these minor limitations to have essentially no impact on the general methodological implications of this study.

### Synthetic metabolic labelling data

During metabolic labelling the nucleoside analogue 4-thiouridine is incorporated into nascent mRNA during Pol II transcription in eukaryotic cells (Miller et al.^12^). This can be utilised to determine population-averaged mRNA production and degradation rates for pools of cells.^12,58^ Anticipating corresponding single-cell application, we implemented an extended rate equation (Supplementary Methods 7) and generated 2D frequency distributions representing the total number of molecules and the number of labelled molecules (Supplementary Figure 10a,b). We directly used deviations regarding these frequency distributions for model fitting since single-cell production and degradation rates cannot be derived using this method because the required mRNA levels at the start of each labelling time interval are unknown. For cell populations, these are estimated from sequentially terminated parallel experiments assuming that the respective population means are identical over time. In addition to the direct use of the 2D frequency distributions, we also calculated population-averaged production and degradation rates mimicking the experimental data and computational approach of Miller et al.^12^ and Sun et al.^58^. In these publications, rates were calculated based on two related exponential growth models. In the present study, we derived alternative formulas based on the above 2D frequency distribution (Supplementary Methods 7). Comparison of these three approaches resulted in quasi equivalent very good performance (e.g., average relative error <5% for *N* = 30; Supplementary Figure 10c).

### Synthetic single-cell tracking data

A more immediate approach to cellular rates is the tracking of individual events in single cells, usually performed based on fluorescence microscopy techniques. Traditionally, such single-cell tracking (SCT) data are employed to identify model parameters by probabilistic likelihood^59^ (Supplementary Methods 8). In addition, we developed a frequentist rate reconstruction method (Supplementary Methods 9). Basically, molecular change and cell growth events were generated using rejection sampling, and total event rates were derived from waiting-time histograms. Subsequently, production, degradation and growth rates were calculated according to their relative event frequencies (Supplementary Figure 11).

### Data availability

The data that support the findings of this study were provided by A. Kashiwagi and T. Yomo (Kashiwagi et al.^2^) and B. Schwalb and P. Cramer (Miller et al.^12^) for the purpose of this study. The data continue to be managed by the original authors. The R and c computer codes generated during the current study are available on request.

## Electronic supplementary material

Supplementary Methods

Supplementary Figures

## Electronic supplementary material

**Supplementary information** accompanies the paper on the *npj Systems Biology and Applications* website (10.1038/s41540-018-0049-0).
