# Supplementary material for: Stochastic system identification without an a priori chosen kinetic model—exploring feasible cell regulation with piecewise linear functions
Source: NPJ Syst Biol Appl. 2018 Apr 11;4:15. doi: 10.1038/s41540-018-0049-0 (PMC5895840; doi:10.1038/s41540-018-0049-0)
Supplement: Supplementary file 1 — Supplementary Methods [file 41540_2018_49_MOESM1_ESM.pdf]

Supplementary Methods

–

Stochastic system identification without an *a priori* chosen kinetic model - exploring feasible cell regulation with piecewise linear functions

Authors

Martin Hoffmann[1,2], Jörg Galle[3]

Affiliations

1. Fraunhofer ITEM, Division of Personalized Tumor Therapy,
BioPark I, Am Biopark 9, 93053 Regensburg, Germany

2. Department of Data Science and Knowledge Engineering, Maastricht University,
Bouillonstraat 8-10, 6211 LH Maastricht, The Netherlands

3. Interdisciplinary Centre for Bioinformatics, University of Leipzig,
Härtelstr. 16-18, 04107 Leipzig, Germany

Supplementary Methods 1 - Stochastic Modelling

The Chemical Master Equation (CME) that corresponds to the rate equation (2) of the main text describes the time evolution of the probability $p(n,i,l)$ for observing $M = 2^l$ cells in cell cycle phase i containing n molecules

$$\frac{dp(n,i,l)}{dt} = \left[E_n^{-1} - 1\right] P(n,i)\, p(n,i,l) + \left[E_n^{+1} - 1\right] D(n,i)\, p(n,i,l) - G(n,i)\, p(n,i,l) + \begin{cases} \sum_{m=n}^{N} G(m,k)\, b(n|m,1/2)\, p(m,k,l-1) & for\ i=1, l \geq 1\ (0\ for\ l=0) \\ G(n,i-1)\, p(n,i-1,l) & for\ i=2,\ldots,k \end{cases} . \quad (4)$$

We do not consider cell density-dependent effects implying that the rates P, D and G are independent of l. The dynamics of the average number of cells $\bar{M}(n,i) = \sum_{l=0}^{\infty} 2^l\, p(n,i,l)$ is given by $d\bar{M}(n,i)/dt = \sum_{l=0}^{\infty} 2^l\, dp(n,i,l)/dt$. Using $\sum_{l=1}^{\infty} 2^l\, p(m,k,l-1) = 2\,\bar{M}(m,k)$ and identifying $\bar{M}(n,i)$ with $F(n,i)$ in (2) proves that the two dimensional rate equation (2) is equivalent to the dynamics of the mean number of cells as conferred by the CME (4).

To characterise the dynamics of molecule production and degradation we neglect cell proliferation and consider a general one-dimensional CME. A second order approximation to a one-dimensional CME is the Fokker-Planck equation (FPE) (Ito form)

$$\frac{dp(x)}{dt} = -\frac{\partial}{\partial x}\left[A(x)\, p(x)\right] + \frac{1}{2}\frac{\partial^2}{\partial x^2}\left[B(x)\, p(x)\right] , \quad (5)$$

with *x* being the continuous representation of the number of molecules *n*. The FPE holds especially well for diffusion type (i.e. single to few step) processes [1] appropriate for the mesoscopic description of transcription and translation that neglects stoichiometric detail. Notably, the FPE coincides with the convection-diffusion equation of fluid dynamics if the diffusion term $B(x) = B$ is constant [2]. The FPE relates to the chemical Langevin equation [3,4] by

$$\frac{dx}{dt} = A(x) + \sqrt{B(x)}\, \zeta , \quad (6)$$

for individual trajectories of *x,* with ς representing standard Gaussian white noise and $A(x)$ and $B(x)$ being denoted as deterministic and noise term, respectively. $B(x)$ is called additive if constant and multiplicative otherwise. According to the definition of $A(x)$ and $B(x)$ (Methods) large noise variations together with a small deterministic term (i.e. noise-driven dynamics) imply high rate correlation while large variations in the deterministic term together with additive noise (i.e. deterministic dynamics) imply high rate anti-correlation.

Supplementary Methods 2 - Mapping between fluorescence intensity and molecule number

The mapping between fluorescence intensity *f* and molecule number *n* was performed according the affine function

$$n = N \frac{f - f_{\min}}{f_{\max} - f_{\min}} \quad , \qquad (7)$$

in which the minimum fluorescence intensity (f_{min}) was determined according to the smallest non-vanishing $\log_{10}$-fluorescence histogram bin and the maximum (f_{max}) was set well beyond the populated range to avoid upper boundary effects. Histograms for different values of *N* were obtained by smoothing-spline interpolation of the experimental histograms.

Supplementary Methods 3 - Noise-Driven Optimization (NDO)

In order to find a maximum number of parameters within the feasible region, we developed the optimization and scouting method 'Noise-Driven Optimization' (NDO). Shortly, trajectories were generated starting from uniformly distributed initial parameters. A single new exploratory parameter set was randomly generated from a previously defined reference parameter set per iteration. Perturbation of individual parameters within the set was performed according to a univariate normal distribution with reference error-dependent standard deviation. This standard deviation was set differently for x- and y-set-points to reflect the smaller parameter space of the ordered x-values. Accordingly, the standard deviation decreased linearly from 0.2 (error=1) to 0.01 (error=0) for x-positions and from 0.3 (error=1) to 0.015 (error=0) for y-positions. All parameters were recorded within the feasible region but new parameter sets became reference parameter sets only if their associated errors were smaller. To reduce parameter space dimension and speed up convergence, x-positions were optimized in pre-screening runs. Final error levels were only marginally affected by this procedure. Performance of NDO was compared to four standard optimization methods (Supplementary Figure 1).

Supplementary Methods 4 - Workflow

This section provides a brief sketch of the main workflow (see also Supplementary Figure 2). Probabilities p_i generally relate to frequencies f_i by $p_i = f_i / \sum_k f_k$.

Data processing 1 (first cytometry dataset)

1) Transformation of the experimental fluorescence intensities to the molecular range $[log(1), log(N+1)]$ according to equation 7 (Supplementary Methods 2).
2) Calculation of histograms and their subsequent least-squares fit by $f(x) = exp(poly3(x))$, with $poly3$ a 3rd order polynomial (Supplementary Figure 3). Fits are adapted for different $N' \leq N$ by spline interpolation while scaling $log(N'+1)$ to $log(N+1)$.
3) Transformation of the above probability distributions for $log(n+1)$ to probability distributions for n according to $p(n) = p_{log}(log(n+1))/(n+1), n = 0 \dots N$.

Parameter identification 1 (first cytometry dataset)

1) Generation of uniformly distributed random initial parameters.
2) Parameter identification according to Noise Driven Optimisation (NDO) using 200 parameter iteration cycles. Evaluation by Mean Absolute Deviation (MAD) regarding the probability distribution after 3 days (slow proliferation, green fluorescence). Simulations were started from the initial distribution (green fluorescence) corresponding to fast proliferation. The same procedure was applied to also obtain parameters reproducing the initial probability distribution. Simulations equilibrated within at most 12h.
3) For graphical display, probability distributions $p(n,i)$, with n the number of molecules and i the cell cycle phase, were obtained by re-simulation with the minimum error parameters saved for each parameter space bin during optimisation (main text). These probabilities were used to calculate population-averaged characteristics such as the mean production rate $\bar{P} = \sum_i \sum_n P(n,i)\, p(n,i)$ or its derivative $\overline{dP} = \sum_i \sum_n dP(n,i)\, p(n,i)$, for which the derivative $dP(n,i)$ regarding n was determined according to spline interpolation for each i.

Data processing 2 (second cytometry dataset)

1) The experimental frequency distributions of the green/red fluorescence log-ratios $F(lgr)$ as provided by Kashiwagi and Yomo were used for identification without processing. To obtain corresponding computational results, simulated frequency distributions for log-green fluorescence were convolved with the initial log-red fluorescence distribution of the first data set (Supplementary Methods 6).
2) For this, simulation results for the frequency distributions of green fluorescent molecules were transformed to frequency distributions for log-green fluorescence using (the inverse of) equation 7 (Supplementary Methods 2) and the frequency/probability log-transformation $F_{log}(log(f)) = f \cdot F(f)$, with f the fluorescence intensity.

Parameter identification 2 (second cytometry dataset)

1) Generation of uniformly distributed random initial parameters for the switch activation condition (depleted medium).
2) Parameter identification was continued for 400 iterations and evaluated by the Mean Absolute Deviation (MAD) regarding the frequency distributions of green/red log-ratios at times 0, 0.5, 2.5, 5 and 7.5h. Parameters were changed from initial (fast proliferation) to switch activation conditions using an explicitly time-dependent induction function (main text). For each set of switch activation parameters (each iteration step), a corresponding set of initial condition parameters was selected from the set of feasible parameters for the initial condition of the first data set according to closest similarity (MAD).
3) For graphical display, the same procedure as for the first cytometry data set was applied per time point (Parameter identification 1).

Data processing 3 (second cytometry dataset, semi-empirical synthetic data)

Using the best fitting (lowest error) parameters identified for the second data set (Parameter identification 2), semi-empirical synthetic reference data were generated using the following methods:

1) Freq 1D: frequency distributions $F(n,i)$, n the number of molecules, i the cell cycle phase.
2) Freq 2D: frequency distributions $F(n,n_l,i)$, n the total number of molecules, n_l the number of labelled molecules, i the cell cycle phase. These data can generally be obtained by single cell evaluation of metabolic labelling experiments.
3) Rates PAV: exact population-averaged rates ($\bar{P},\bar{D},\bar{G}$) as obtained from the original rates $P(n,i)$, $D(n,i)$ and $G(n,i)$ and the simulated probabilities $p(n,i)$. These rates can be well estimated using metabolic labelling experiments (Parameter identification 1).
4) Rates SCT id: original rates $P(n,i)$, $D(n,i)$ and $G(n,i)$ for $i=1$ when concentration equals the number of molecules (main text). This corresponds to ideal results of single cell tracking (infinite data).
5) Rates SCT: rates $P(n,i)$, $D(n,i)$ and $G(n,i)$ for $i=1$ (concentration=number of molecules) as estimated from simulated single cell trajectories by the histogram method (Supplementary Methods 9, concentration used for reconstruction). Rates may be undefined at some n because of too few events (minimum 100 required) and may later remain un-evaluated at these points. For better comparison, these states were also not evaluated for Rates SCT id in 4).
6) LogLike SCT: negative log-likelihood of the synthetic single cell tracking data (same data as used for Rates SCT in 5) (Supplementary Methods 8).

These data were provided per 0.5h interval starting from 2h onwards, i.e. first interval 2.0-2.5h, last interval 7.0-7.5h (main text, Supplementary Methods 10).

Parameter evaluation 3 (semi-empirical synthetic data)

For comparison with the reference data defined above (Data processing 3), all parameters of the feasible region (minimum error parameters per parameter space bin) were evaluated against the reference parameters using the following metrics:

1) Freq 1D: sum of absolute differences regarding $F(n,i)$, scaled by twice the sum of the experimental histograms, taken across all times.
2) Freq 2D: sum of absolute differences regarding $F(n,n_l,i)$, scaled by twice the sum the of experimental histograms, taken across all times.
3) Rates PAV: scaled mean absolute deviation (sMAD) of the exact population-averaged rates $\bar{P},\bar{D}$ and $\bar{G}$ taken across all three rates and all times. Scaling refers to the absolute rate limits: 12/h for P,D and 0.5 · ln(2)/h for G.
4) Rates SCT id: scaled mean absolute deviation (sMAD) of the original rates rates $P(n,i)$, $D(n,i)$ and $G(n,i)$ for $i=1$ (concentration=number of molecules) across all three rates and all times.
5) Rates SCT: scaled mean absolute deviation (sMAD) of the original rates $P(n,i)$, $D(n,i)$ and $G(n,i)$ for $i=1$ as compared to the reference data obtained by reconstruction from simulated single cell trajectories.
6) LogLike SCT: negative log-likelihood of the synthetic single cell tracking data evaluated for all parameter sets (Supplementary Methods 8).

Note that 1)-4) refer to ideal measurement, i.e. simulated data were directly used for error calculation, while 5)-6) involve finite sampling from random trajectories (synthetic data).

Data processing 4 (metabolic labelling data)

1) Metabolic labelling data, consisting of mRNA expression values (T), production rates (P) and half-lifes ($\tau_{1/2}$) for times 0, 6, 12, …, 36 minutes, were quality-filtered according to the original annotation provided by Miller et al. and completeness, i.e. no missing values. This resulted in 2065 genes showing complete time series. Half-lifes were transformed to degradation rates according to $D = T \cdot ln(2)/\tau_{1/2}$ (Supplementary Methods 7), assuming that the normalised microarray expression values T can substitute for the number of molecules per cell.
2) Individual time series were interpolated by smoothing splines (smoothing parameter = 0.1) and up-sampled minute-wise (0, 1, 2, …, 36 minutes). Time derivatives were analogously determined from the same interpolating splines.

Data evaluation 4 (metabolic labelling data)

1) Correlation between the time-series for T, P, D and their derivatives were based on the above interpolated time series data. 2065 correlation values were obtained per pairwise comparison and presented as empirical probability distributions (Figure 5g-h).
2) Reference distributions were obtained by evaluating all 7! = 5040 time-point permutations applied to one of the two time-series compared.
3) For obtaining linear trends, the interpolated time series were respectively fitted by a linear (affine) function. Positive linear slope was classified as positive trend, negative slope as negative trend.
4) For Figure 5k, time series for T, P and D were gene-wise standardised (mean=0, standard deviation=1). Genes were clustered according to complete linkage hierarchical clustering regarding T and each cluster was evaluated for standard deviation of T, P and D. Subsequently, these values were ploted as a function of the linear slope (trend) of the cluster mean regarding T.

Supplementary Methods 5 - Population-weighted rate correlation

Correlation of production and degradation rates as displayed in Figures 2 and 3 was population-weighted in order to concentrate assessment on the relevant (i.e. populated) range according to

$$cor(P,D) = \frac{1}{k}\sum_i \left(\frac{\sum_n p(n,i)\big(P(n,i)-\bar{P}(i)\big)\big(D(n,i)-\bar{D}(i)\big)}{\sqrt{\sum_n p(n,i)\big(P(n,i)-\bar{P}(i)\big)^2}\sqrt{\sum_n p(n,i)\big(D(n,i)-\bar{D}(i)\big)^2}} \right) , \qquad (8)$$

in which $p(n,i)$ is the probability distribution of the number of molecules n and cell cycle phases i. $P(n,i)$ and $D(n,i)$ denote the production and degradation rates, respectively, and $\bar{P}(i)$ and $\bar{D}(i)$ their means across n. Thus, $cor(P,D)$ is the average of population-weighted correlations across cell cycle phases.

Supplementary Methods 6 - Log-ratios for dynamic fluorescence cytometry data

For the calculation of green-to-red log-ratios, the distribution of red fluorescence was calculated from the equilibrium distribution at fast proliferation (Supplementary Figure 3). The dynamics of green branch activation were simulated based on integration of equation (2) analogous to the main text section leading to Figure 2. The resulting frequency distributions $f_X(x)$ were log-transformed according to $f_{\log X}(\log x) = x\, f_X(x)$ and convolved with the distribution for red fluorescence assumed to be independent of time. In the second experiment, some promoter imbalance was present as evidenced by the non-zero-centred distributions at 0.5h in Figure 3A of [5] (also confirmed by personal correspondence). This effect was accounted for by somewhat downshifting the red fluorescence distribution since this fitted the data much better than a corresponding green fluorescence upshift suggesting that the imbalance was caused by reduced *trc*-promoter activation.

Supplementary Methods 7 - Rate approximations for dynamic labelling experiments

Production and degradation rates estimated as described below derive from the total number of molecules T, the number of labeled molecules L and the number of cells M measured over time. These values are generally obtained by destructive measurment of sequentially terminated experiments making this approach accessible only for cell populations. Miller et al. [6] assumed exponential growth of the total number of molecules and exponential decay of the number of unlabeled molecules. Their basic model reads

$$\begin{aligned} T &= T_0 \exp(\sigma t) \\ L &= \lambda T_0 \left[\exp(\sigma t) - \exp(-\rho t) \right] \end{aligned} \qquad (9)$$

with λ the labeling efficiency, the growth and decay rates

$$\begin{aligned} \sigma &= \frac{d \log(T(t_l))}{dt} \\ \rho &= -\sigma - \frac{1}{t_l} \log\left(1 - \frac{\Lambda(t_l)}{\lambda} \right) \end{aligned} \quad , \qquad (10)$$

respectively, t_l the labeling time and $\Lambda = L/T$ the fraction of labeled molecules. The rates σ and ρ are effective global rates. Average cellular production and degradation rates are calculated according to

$$\begin{aligned} \overline{P} &= \bar{n}\ (\sigma + \rho) \\ \overline{D} &= \bar{n}\ \rho \end{aligned} \quad , \qquad (11)$$

in which $\bar{n} = T/M$ is the average number of molecules per cell. We added the second equation since Miller et al. did not explicitly calculate $\overline{D}$. In [6] $\bar{n}$ is determined proportional to the relative expression of each gene assuming a constant total number of mRNAs per cell. This approach is inapplicable in the present study considering a single molecular species. However, equivalence to [6] is evident from the molecular half-life $\tau_{1/2} = ln(2)/\rho = T\, ln(2)/(M\overline{D})$. Sun et al. [7] differ in that they calculate

$$\begin{aligned} \rho &= -\frac{1}{t_l} \log\left(\frac{T(t_l) - L(t_l)/\lambda}{T(0)} \right) \\ \overline{P} &= \frac{\bar{n}\ (\sigma + \rho)\ L(t_l)}{\lambda\ [\exp(\sigma t_l) - \exp(-\rho t_l)]} \end{aligned} \quad . \qquad (12)$$

Our own approach makes use of the rate equation for the 2D frequency distribution $F(n, n_l)$. We define $T = \sum_n n\, F(n)$ and $L = \sum_n n_l\, F_l(n_l)$, with $F(n)$ and $F_l(n_l)$ the respective marginal frequency distributions of $F(n, n_l)$. Changes in T and L obey

$$\frac{dT}{dt} = \sum_n n\left(\frac{dF^e(n)}{dt}\right)$$
$$\frac{dL}{dt} = \sum_{n_l} n_l\left(\frac{dF_l^e(n_l)}{dt}\right) \quad . \qquad (13)$$

The 2D rate equation for expression(e)-related change reads

$$\frac{dF^e(n,n_l)}{dt} = \lambda\,\alpha(n-1)\,F(n-1,n_l-1) + (1-\lambda)\,\alpha(n-1)\,F(n-1,n_l) - [\alpha(n)+\delta(n)]F(n,n_l)$$
$$+[1-p_l(n+1,n_l)]\delta(n+1)\,F(n+1,n_l) + p_l(n+1,n_l+1)\,\delta(n+1)\,F(n+1,n_l+1) \quad , \qquad (14)$$

in which $p_l(n,n_l) = n_l/n$ is the probability for a labeled molecule in bin (n,n_l) (Supplementary Figure 8). Using $P(N) = D(0) = 0$ results in

$$\frac{dT}{dt} = M\left(\overline{P} - \overline{D}\right)$$
$$\frac{dL}{dt} = M\left(\lambda\,\overline{P} - E[\,p_l\,D\,]\right) \qquad (15)$$

in which $E[\,\lambda\,P] = \lambda\,\bar{P}$ and $E[\,p_l\,D]$ are the expected production and degradation rates of labeled molecules. For a closed solution of (15) $E[\,p_l\,D]$ must be expressed in terms of the macroscopic variables. A natural choice is $E[\,p_l\,D] = \mu\,\overline{D}$ with $\mu = c_\mu\,L/T$. Assuming c_μ to be constant leads to

$$\overline{P} = \frac{1}{M(\lambda-\mu)}\left[\frac{dL}{dt} - \mu\,\frac{dT}{dt}\right]$$
$$\overline{D} = \frac{1}{M(\lambda-\mu)}\left[\frac{dL}{dt} - \lambda\,\frac{dT}{dt}\right] \quad . \qquad (16)$$

Evaluating equations (16) at the start of labelling ($L = 0$) would allow using $\mu = 0$ not requiring a specific value for c_μ. However, in practice numerical evaluation involves finite differences and values of $c_\mu \sim 0.5$ or smaller (due to exponential growth) can be expected to be appropriate (Supplementary Figure 10c). The formulas of this section straightforwardly generalize to the presence of multiple cell cycle phases (additional summation).

Supplementary Methods 8 - Likelihood calculation

Likelihoods were derived according to Wilkinson [8] and efficiently calculated using event histograms obtained from multiple cell trajectories. According to Wilkinson the likelihood of the observational event data of a single trajectory is given by

$$L = \left\{ \prod_{i=1}^{n-1} h_{\nu_i}(c_{i-1}) \exp\left(-h_0(c_{i-1})\,(t_i - t_{i-1})\right) \right\} \exp\left(-h_0(c_{n-1})\,(t_n - t_{n-1})\right) \quad (17)$$

with $c_i = c(t_i)$ the concentration at time t_i, the hazard $h_0 = h_P+h_D+h_G$ the sum of production (h_P), degradation (h_D) and growth (h_G) rates and ν_i the index associated with the event at t_i (i.e. either *P, D* or *G*) (h_P, h_D and h_G coincide with the rates *P, D* and *G* of the main text). Notably, we treated the growth rate analogous to molecule production and degradation rates and defined the time point vector as $t = (t_0, t_1, \ldots, t_{n-1}, t_n)$, in which t_0 and t_n are the start and end time points not necessarily associated with events. In our simulations, concentrations c_i and waiting times $\tau_i = t_i - t_{i-1}$ were discretised and histograms $H_0[\tau,c]$, $H_E[\tau,c]$, $H_P[c]$, $H_D[c]$ and $H_G[c]$ for total, end, production, degradation and growth events, respectively, were calculated from multiple cell trajectories. Specifically, $H_0[c] = \Sigma_\tau H_0[\tau,c] = \Sigma_\nu H_\nu[c]$ for $\nu = P,D,G$ is valid. For given event rates h_P, h_D and h_G the log-likelihood function is finally given by

$$\ln(L) = \sum_{\nu,c} H_\nu[c] \ln h_\nu(c) - \sum_{\tau,c} H_0[\tau,c]\, h_0(c)\,\tau - \sum_{\tau,c} H_E[\tau,c]\, h_0(c)\,\tau$$

with c and τ running across all concentrations and waiting times, respectively. Evaluation was restricted to concentrations with $H_0[c] \geq 100$.

Supplementary Methods 9 - Histogram-based rate reconstruction from single cell tracking data

Reconstruction of production, degradation and growth rates from simulated trajectories was performed along the same lines as the likelihood calculation above. Basically, total event waiting time histograms stratified according to discrete lower integer concentrations were used to fit exponential distributions and derive average concentration-dependent total event rates, i.e. $h_0(c)$ was calculated by fitting exponential distributions to each column of $H_0[\tau,c]$. Relative frequencies of production, degradation and growth (i.e. cell cycle progression) events were then used to calculate the respective final rates according to relative event numbers, i.e. $h_\nu(c) = h_0(c)\, H_\nu[c]/H_0[c]$ for $\nu = P,D,G$. This procedure was applied to sequential 0.5h time intervals (Supplementary Figure 11). Multiple cell cycle phases required up-scaling of the nominal growth rate during simulation. While this is straightforward for rejection sampling, exact scaling appeared involved for Gillespie's Stochastic Simulation Algorithm (SSA), even more so for its time-dependent variant [9] (response induction). As a consequence, we used fixed time-step rejection sampling for the generation of synthetic single cell trajectories. The integration time step was 1 minute throughout the study and equalled the sampling time step of cell tracking. The starting population consisted of 50,000 cells. Only histogram bins containing at least 100

events were evaluated. The generated data were also used for likelihood calculation. Note that if promoter switching is relevant, as e.g. in the model of Kepler and Elston [10], corresponding adjustments in histogram identification (on/off states) are required.

Supplementary Methods 10 - Error calculation

Errors for parameter fits were calculated as sum of absolute deviations from the experimental fluorescence intensity data normalized by twice the sum of the respective experimental histogram values, which is a strict error bound for probability distributions (Figures 1 and 2) and an approximate error bound for frequency distributions (Figure 3). Errors and likelihoods for the method comparison (Figure 5) only include times larger than 2h stratified according to 0.5h intervals, i.e. first interval 2.0-2.5h, … , last interval 7.0-7.5h, to exclude effects of more ambiguous parameter identification during fast proliferation (incomplete induction). Rate errors and likelihoods refer to production, degradation and growth rates. Deviations in response induction are treated implicitly by the above time stratification. Rates were scaled by their range before mean absolute deviation (MAD) errors were calculated across rates. Rates reconstructed from single cell tracking (SCT) data (Rates SCT) were evaluated only for concentrations for which the event number was at least 100. For better method comparison, this was also applied to the corresponding ideal method (Rates SCT id). As a consequence, Rates SCT id errors can be as low as Rates SCT errors even at high parameter distances (Supplementary Figure 12e). For standard fitting problems (e.g. rational fits to feasible region coverage, exponential fits to waiting time histograms) standard least squares procedures were employed.

Supplementary Methods 11 - Eigenvalues and Eigenvectors in Hessian sensitivity analysis

Data were restricted to the lower distance quartile (i.e. distances up to ~0.6). Hessian sensitivity analysis was performed recursively (20 iterations) as this slightly reduced negativity of Eigenvalues. Occasionally occurring negative Eigenvalues were due to skewed data structures and not to saddle points (verified by manual inspection of projections to the main axes). The y-set-point IY1 was omitted from sensitivity analysis for this reason (narrow and skewed distribution close to the lower boundary).

References

1 Grima, R., Thomas, P. & Straube, A. V. How accurate are the nonlinear chemical Fokker-Planck and chemical Langevin equations? *J Chem Phys* **135**, 084103, doi:10.1063/1.3625958 (2011).

2 Ao, P. Global view of bionetwork dynamics: adaptive landscape. *J Genet Genomics* **36**, 63-73, doi:10.1016/S1673-8527(08)60093-4 (2009).

3 van Kampen, N. G. *Stochastic processes in physics and chemistry.*, (Elsevier, Amsterdam, 2004).
4 Gillespie, D. T. The chemical Langevin equation. *The Journal of Chemical Physics* **113**, 297-306 (2000).
5 Kashiwagi, A., Urabe, I., Kaneko, K. & Yomo, T. Adaptive response of a gene network to environmental changes by fitness-induced attractor selection. *PLoS One* **1**, e49, doi:10.1371/journal.pone.0000049 (2006).
6 Miller, C. *et al.* Dynamic transcriptome analysis measures rates of mRNA synthesis and decay in yeast. *Molecular systems biology* **7**, 458, doi:10.1038/msb.2010.112 (2011).
7 Sun, M. *et al.* Comparative dynamic transcriptome analysis (cDTA) reveals mutual feedback between mRNA synthesis and degradation. *Genome Res* **22**, 1350-1359, doi:10.1101/gr.130161.111 (2012).
8 Wilkinson, D. J. *Stochastic Modelling for Systems Biology*. (Chapman & Hall / CRC, Boca Raton, 2006).
9 Lu, T., Volfson, D., Tsimring, L. & Hasty, J. Cellular growth and division in the Gillespie algorithm. *Systems biology* **1**, 121-128 (2004).
10 Kepler, T. B. & Elston, T. C. Stochasticity in transcriptional regulation: origins, consequences, and mathematical representations. *Biophysical journal* **81**, 3116-3136, doi:10.1016/S0006-3495(01)75949-8 (2001).
