# Supplementary material for: Stochastic system identification without an a priori chosen kinetic model—exploring feasible cell regulation with piecewise linear functions
Source: NPJ Syst Biol Appl. 2018 Apr 11;4:15. doi: 10.1038/s41540-018-0049-0 (PMC5895840; doi:10.1038/s41540-018-0049-0)
Supplement: Supplementary file 2 — Supplementary Figures [file 41540_2018_49_MOESM2_ESM.pdf]

Supplementary Figures

–

Stochastic system identification without an *a priori* chosen kinetic model - exploring feasible cell regulation with piecewise linear functions

Authors

Martin Hoffmann[1,2], Jörg Galle[3]

Affiliations

1. Fraunhofer ITEM, Division of Personalized Tumor Therapy,

BioPark I, Am Biopark 9, 93053 Regensburg, Germany

2. Department of Data Science and Knowledge Engineering, Maastricht University,

Bouillonstraat 8-10, 6211 LH Maastricht, The Netherlands

3. Interdisciplinary Centre for Bioinformatics, University of Leipzig,

Härtelstr. 16-18, 04107 Leipzig, Germany

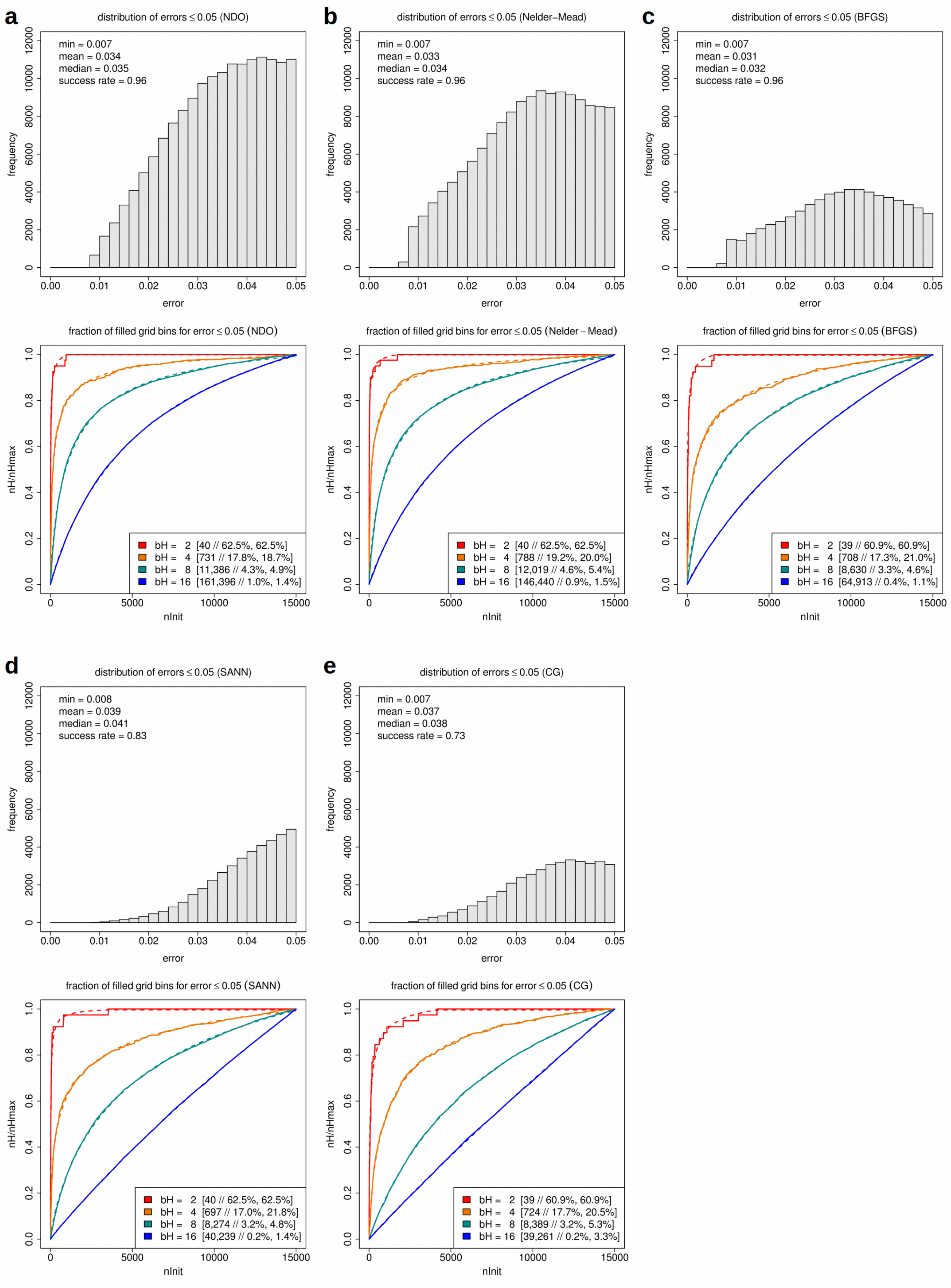

Supplementary Figure 1. Frequency distribution of data fitting errors and mapping of the feasible region. **a** Top: Distribution of errors ≤ 0.05 for noise-driven optimization (NDO). Minimum, median and mean errors as well as success rates are given in the top left corner. Success rates are the fraction of

optimization runs reaching the feasible region defined by an error ≤ 0.05. Bottom: Scaled number of occupied grid bins (nH/nHmax) in the 6-dimensional (6D) parameter space (y-set-points) as a function of the number of successfully initiated searches (nInit). The parameter space is subdivided into bH=2, 4, 8, and 16 bins per dimension, respectively. A 6D-bin is considered occupied if entered at least once during the 200 iterations per random run. For bH=2 saturation is reached early (exhaustive search), while for bH=16 the feasible region is still incompletely covered after 15,000 random initializations. Data are fitted by rational functions as suggested by stochastic integration theory. This allows estimation of asymptotic feasible region size. The bracketed numbers in the legend state the maximum number of filled bins and the percentages of the actually filled and asymptotically saturated bins relative to all bH^6 available bins. This indicates that NDO searches were exhaustive by 100, 95, 88, and 71% for bH=2, 4, 8, and 16, respectively. The scaling of occupied bins with nInit was almost exactly polynomial with exponent 4.6 (fractal box counting dimension). **b**-**e** Analogous results for the Simplex (Nelder-Mead) (**b**), BFGS (Broyden-Fletcher-Goldfarb-Shanno) (**c**), Simulated Annealing (SANN) (**d**) and Conjugated Gradient (CG) (**e**) algorithms. For BFGS, the high success rate (0.96) goes along with relatively few occupied bins (40% of NDO) indicating that BFGS trajectories tend to follow common paths. All reference methods (**b**-**e**) according to the optim R-package. Scaling $N = 30$. Results for $N = 15$ and 60 were consistent. Original data by courtesy of A. Kashiwagi and T. Yomo (first data set, slow proliferation).

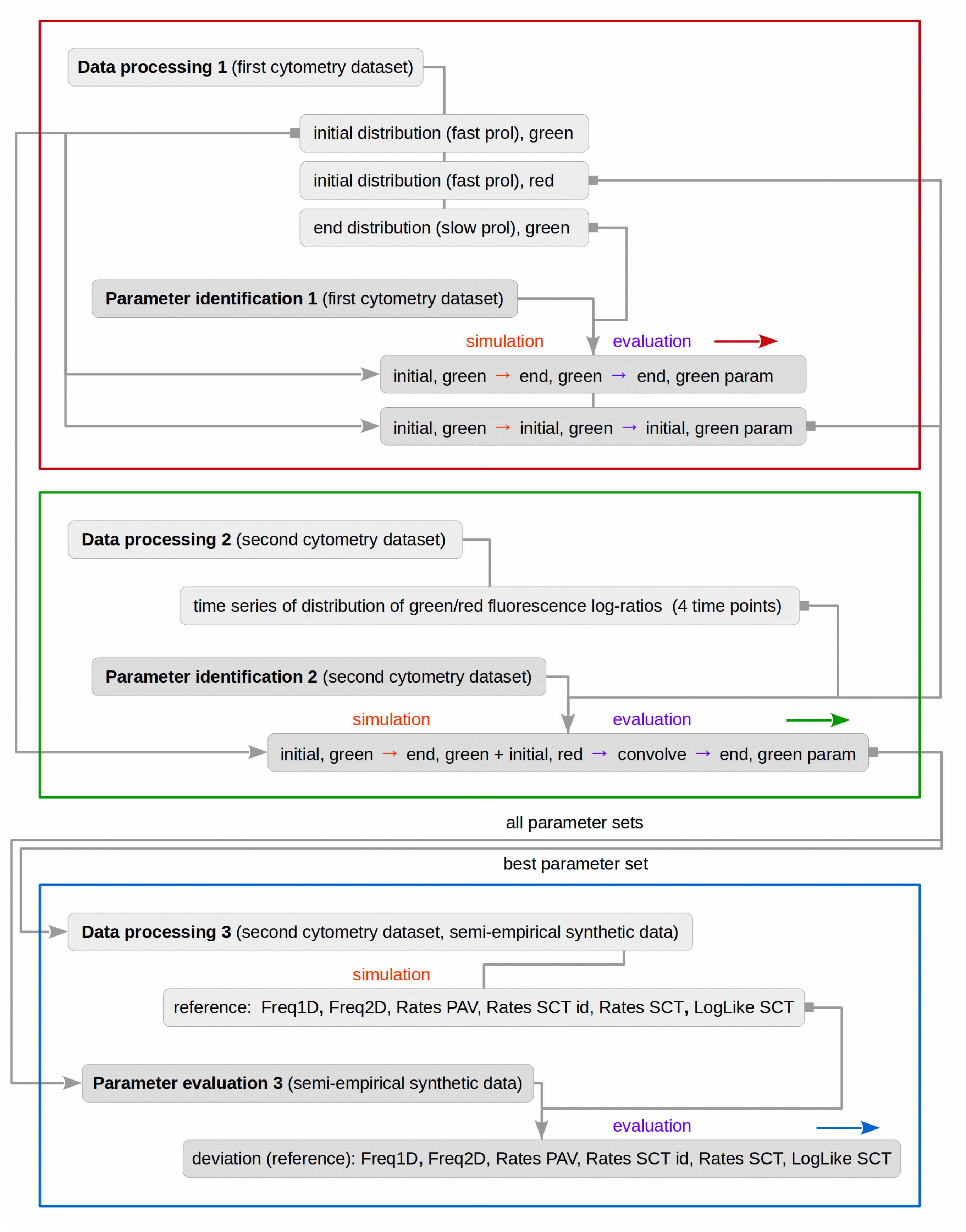

Supplementary Figure 2. Workflow diagram. **Data processing 1** (first cytometry dataset): initial (fast proliferation) and end (slow proliferation) distributions of green and red molecules are prepared from the original data. **Parameter identification 1** (first cytometry dataset): starting from the initial distribution of green molecules (initial, green), simulations for the end distribution of green molecules (end, green) are performed. Comparing simulated and experimental data (end, green) for low errors results in feasible parameters (end, green param). The same procedure is applied for the initial

distribution of green molecules (initial, green) to generate feasible model representations of this initial distribution (initial, green param) required for Parameter identification 2. **Data processing 2** (second cytometry dataset): time series of the distribution of green/red fluorescence log-ratios at 4 times: 0, 0.5, 2.5, 5 and 7.5h, provided as original data. **Parameter identification 2** (second cytometry dataset): starting from the initial distribution of green molecules (initial, green; first dataset) simulations were performed for the end distribution of green molecules (end, green). These data were log-transformed and convolved with the log-transformed initial red florescence data of the first dataset (initial, red; first dataset). The resulting green/red log-ratios were compared to the experimental data for low errors resulting in feasible parameters (end, green param). The simulation used an explicitly time dependent transfer between initial and end conditions (induction) thus requiring both initial and end parameters. While end parameters are determined as stated above, a best matching initial parameter set from the first data set (initial, green param) is selected for each end parameter set. **Data processing 3** (second cytometry dataset, semi-empirical synthetic data): the best fitting parameter set from parameter identification 2 is selected for the generation of different semi-empirical synthetic reference data of different types: Freq 1D, Freq2D, Rates PAV, Rates SCT id, Rates SCT and LogLike SCT. **Parameter evaluation 3** (semi-empirical synthetic data): The same procedure as in Data processing 3 is applied to all feasible parameters resulting from Parameter identification 2, except for Rates SCT, for which the original model rates were used instead of reconstructed rates. Subsequently, deviations regarding the corresponding reference data were evaluated. The red, green and blue arrows indicate result output. For details see Supplementary Methods 4.

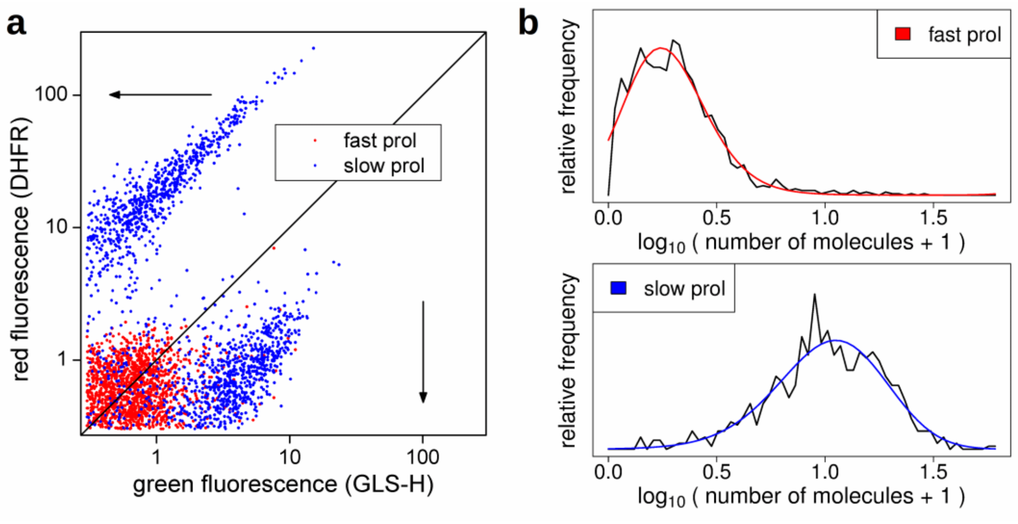

Supplementary Figure 3. Fluorescence cytometry data of Kashiwagi et al. and split into two sub-populations. **a** Scatterplot of green versus red fluorescence intensities. During fast proliferation (red points) expression of the respective reporter proteins for the enzymes GLS-H and DHFR is low due to molecular dilution. During slow proliferation (blue points) switching results in high expression of only one reporter. Slow proliferation data points below the diagonal were projected to the x-axis and those above it to the y-axis (arrows) to form two independent one-dimensional data sets for each sub-population. Some intensity spillover between fluorescence channels is evident from the diagonal direction of the blue point clouds. According to Roederer [1] further major sources of inaccuracy are channel binning and photon-counting errors. **b** Histograms for the fast and slow proliferation populations as projected to the x-axis and the corresponding fitted empirical density functions (Supplemenary Methods 4) are shown as a function of the number of molecules ($N = 60$) (Supplemenary Methods 2). Original data by courtesy of A. Kashiwagi and T. Yomo (first data set).

1. Roederer, M. Spectral compensation for flow cytometry: visualization artifacts, limitations, and caveats. *Cytometry* 45, 194-205 (2001).

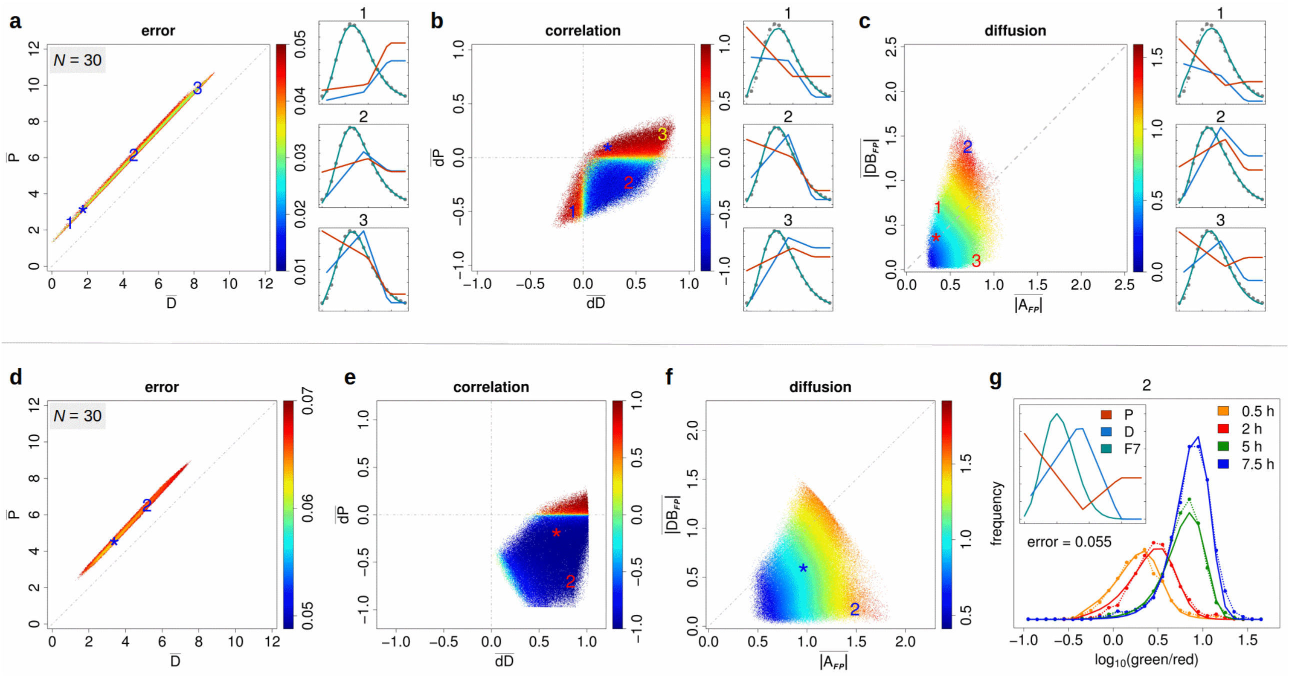

Supplementary Figure 4. a-g Results for $N = 30$ analogous to those for $N = 15$ and $N = 60$ displayed in Figure 2 (**a-c**) and Figure 3 (**d-g**). See Figures 2 and 3 for notation.

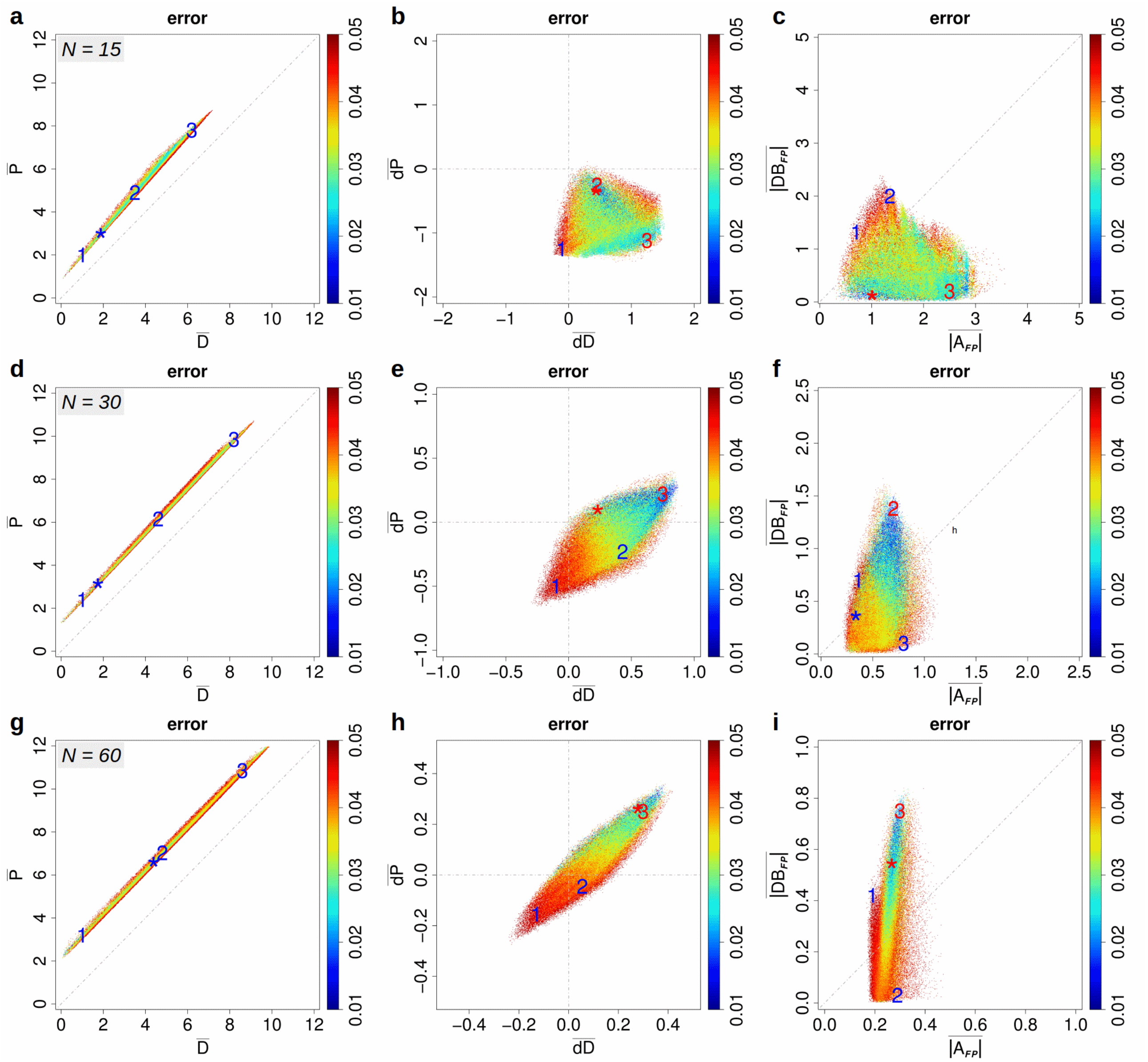

Supplementary Figure 5. Data fitting error as a function of different rate function projections. **a-i** Graphics analogous to Figure 2, however, with the mid and right columns (**b**,**e**,**h**; **c**,**f**,**i**) showing the error (colour-coded) in place of correlation and diffusion, respectively. Results for $N = 15$ (**a-c**), $N = 30$ (**d-f**) and $N = 60$ (**g-i**). See caption of Figure 2 for notation.

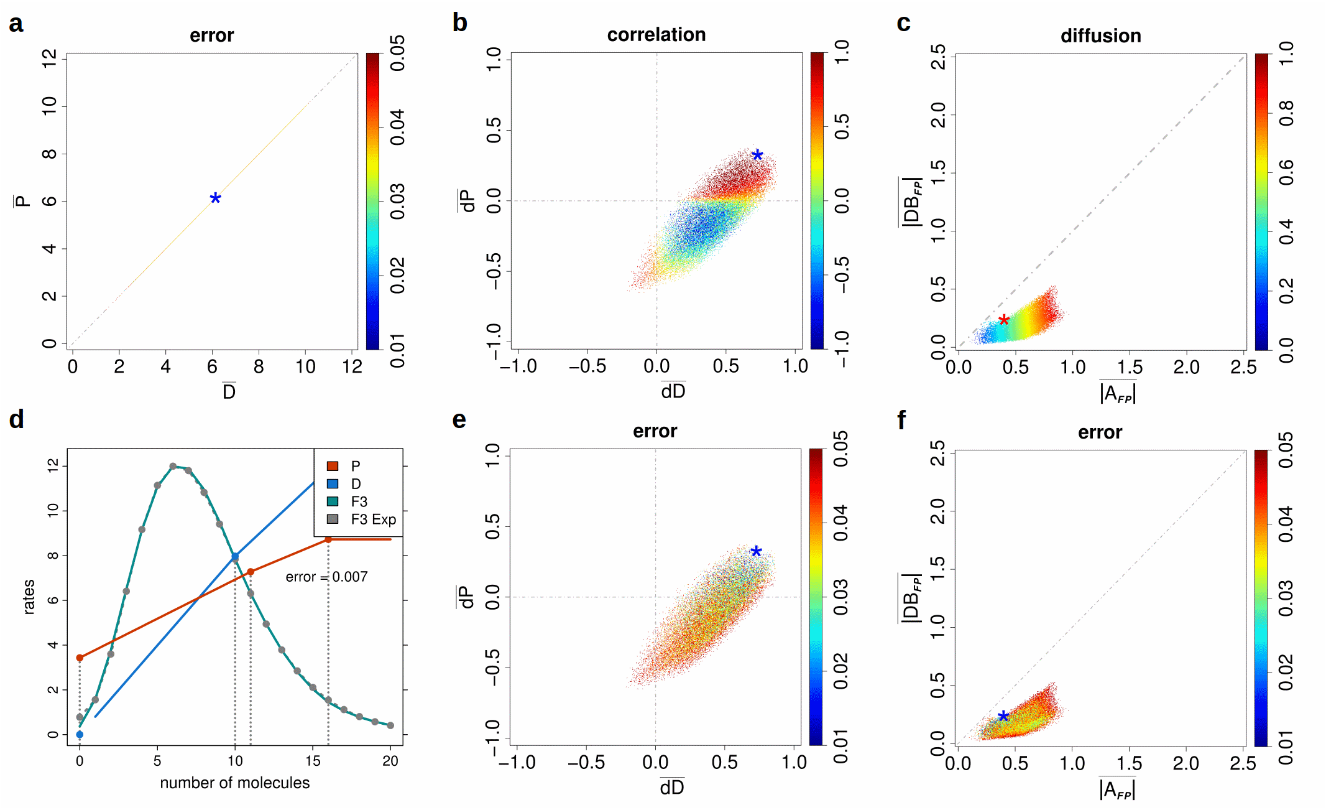

Supplementary Figure 6. Results for assumed zero proliferation for $N = 30$. **a** Population-averaged production ($\overline{P}$) and degradation ($\overline{D}$) rates equal each other as expected. **b** The feasible region (error ≤ 0.05) in terms of population-averaged production ($\overline{dP}$) and degradation ($\overline{dD}$) rate derivatives is similar to the original proliferation case (Supplementary Figure 4e). **c** The feasible region is almost completely confined to dominant deterministic dynamics as compared to proliferating cells (Supplementary Figure S5f). The same applies if the simulation (here started from fast proliferation equilibrium) is initiated from a delta distribution-like peak placed at the mean of the final slow proliferation equilibrium (in this case strictly no deterministic term is required for shifting the mean). **d** Production (P) and degradation (D) rates and the simulated ($F3$) and experimental ($F3\ Exp$) frequency distributions for minimum error parameters. **e**-**f** Analogous to panels **b**-**c** but showing the error (colour-coded) in place of correlation and diffusion, respectively. The asterisk (*) indicates the error minimum.

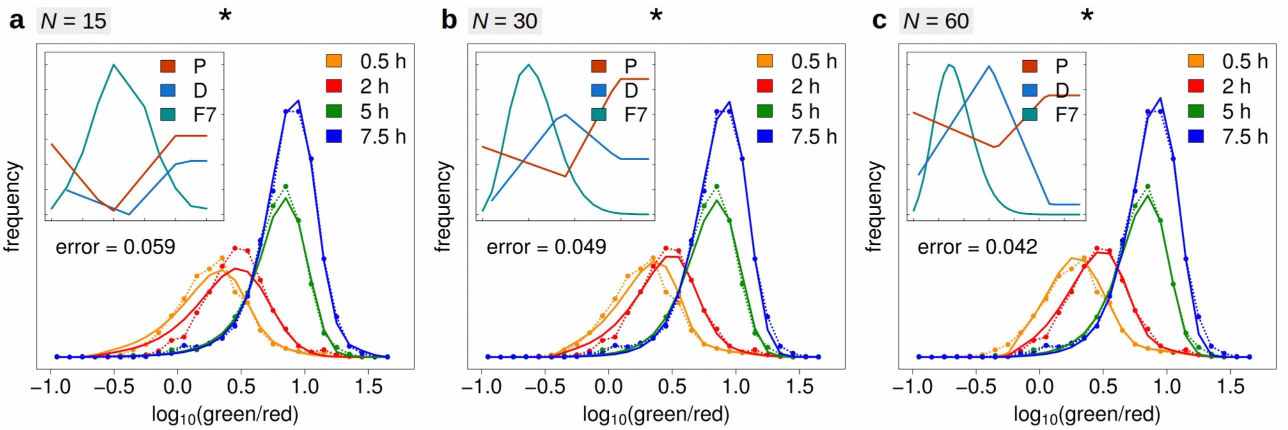

Supplementary Figure 7. Experimental and fitted distributions of green/red fluorescence log-ratios for the minimal error states. **a** Plot analogous to Figure 3g ($N = 15$) but for parameters corresponding to the minimum error (*). **b** Analogous to Supplementary Figure 4**g** ($N = 30$) but for minimum error parameters. **c** Plot analogous to Figure 3**k** ($N = 60$) but for minimum error parameters.

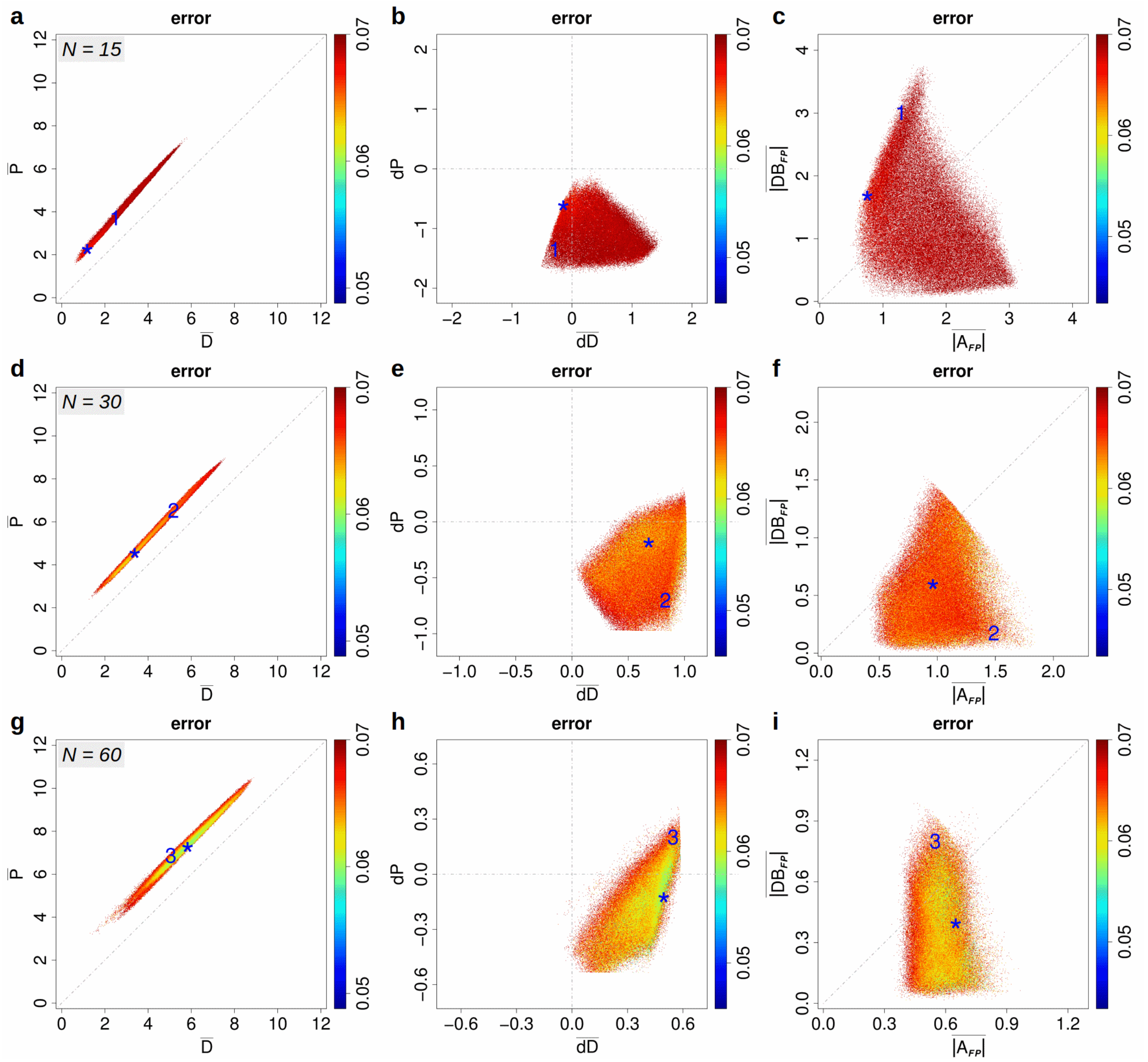

Supplementary Figure 8. Data fitting error as a function of different rate function projections. **a**-**i** Graphics analogous to Figure 3**d**-**f**,**i**-**k** of the main text, however, with the middle and right columns (**b**,**e**,**h**; **c**,**f**,**i**) showing the error (colour-coded) in place of correlation and diffusion, respectively. Results for $N = 15$ (**a**-**c**), $N = 30$ (**d**-**f**) and $N = 60$ (**g**-**i**), respectively. See caption of Figure 3 for notation.

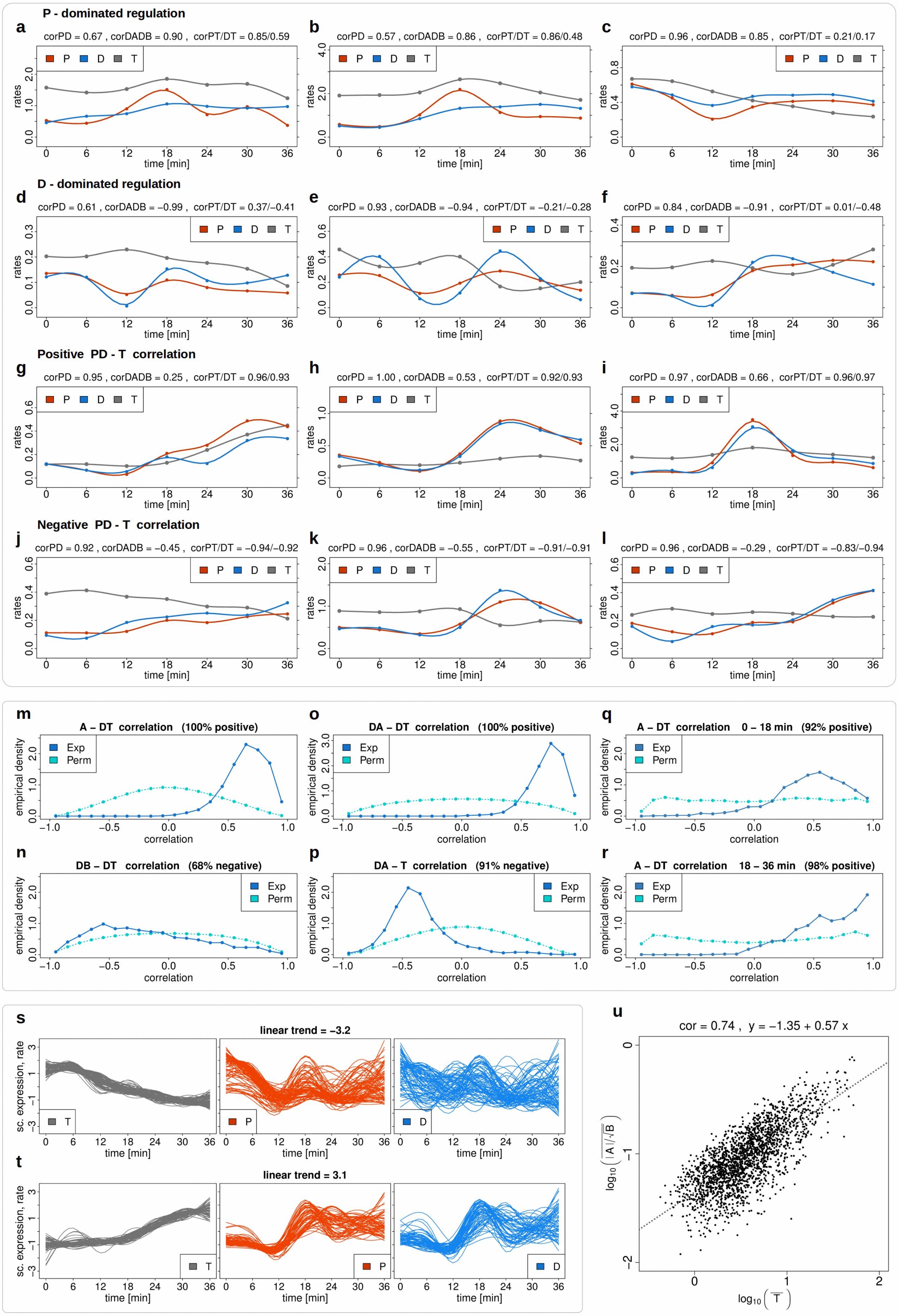

Supplementary Figure 9. Additional graphics for the metabolic labelling data on yeast osmotic stress response. **a**-**l** Three further examples for P(**a**-**c**)- and D(**e**-**g**)-dominated regulation, as well as positive (**g**-

i) and negative (**j**-**l**) correlation between (well correlated) P and D and the expression level T. **m** Entirely positive correlation between deterministic term A and time derivative DT of T. **n** Tendency towards negative correlation between time derivative DB of B and DT. **o** Exclusively positive correlation between time derivatives DA of A and DT. **p** Strongly negative correlation between DA and T suggests stabilising regulation for T. **q**-**r** The association between A and DT becomes stronger over time (**q**: 0-18 min; **r**: 18-36 min). **s**-**t** Time courses of T, P and D for clusters with extremely negative (**s**) and extremely positive (**t**) linear expression trend (cf. Figure 4). **u** Signal-to-noise ratio: log-log plot of the time-averaged signal-to-noise ratio $A/\sqrt{B}$ according to the Langevin equation (Supplementary Methods 1) versus time-averaged T. The signal-to-noise ratio increases with expression level as expected. The linear slope of 0.57 indicates an approximate square root dependence. Accordingly, stochastic effects are most relevant at low molecule numbers. See Figure 4 for notation.

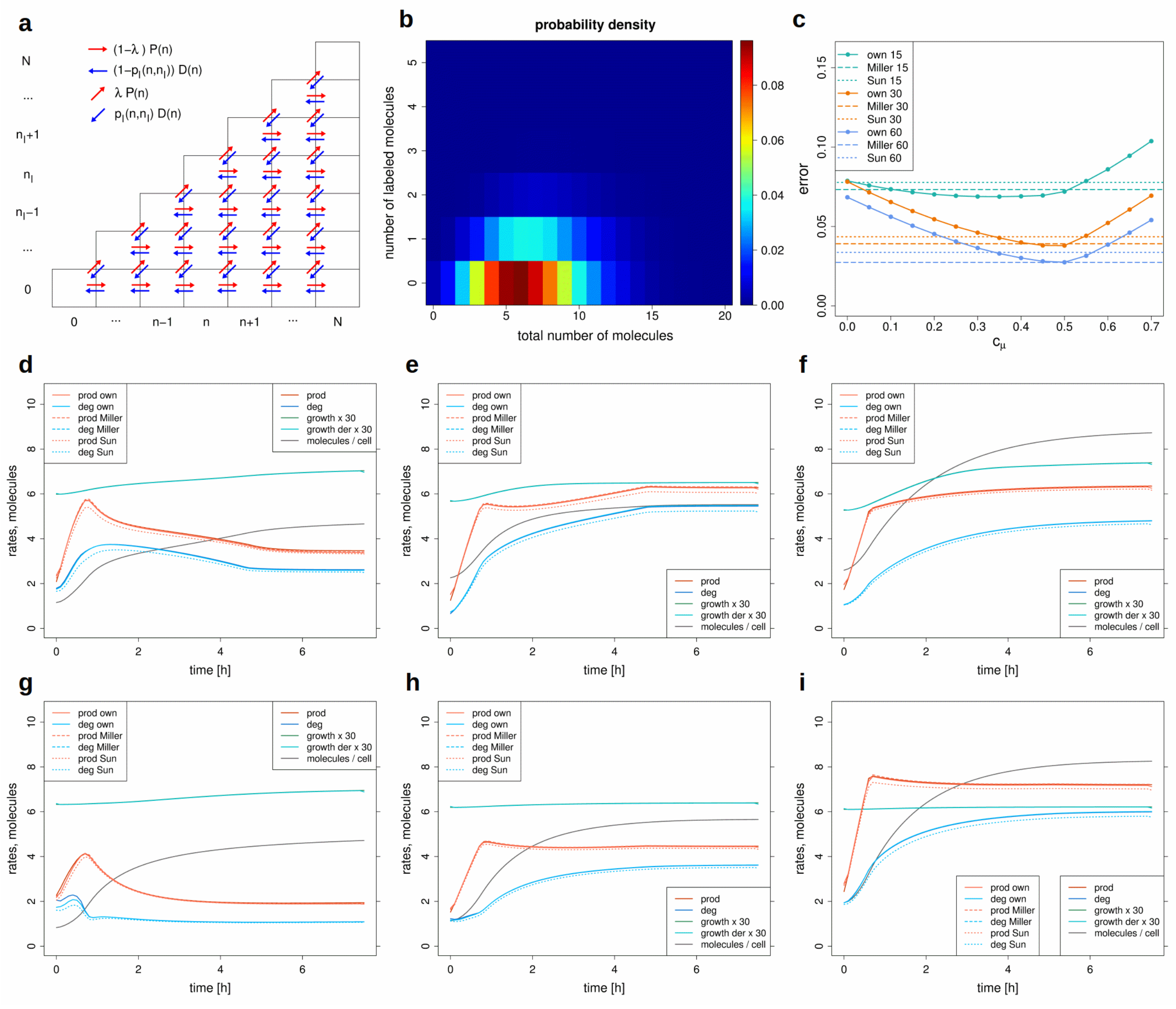

Supplementary Figure 10. Modelling of single cell metabolic labelling. **a** Schematic of the 2D rate equation (Supplementary Methods 7) for the total number of molecules (n) and the number of labeled molecules (n_l). Production and degradation steps are represented by red and blue arrows respectively. λ denotes the labelling efficiency, $p(n, n_l) = n_l/n$ the probability for a labelled molecule, and $P(n)$ and $D(n)$ the production and degradation rates, respectively. **b** Example of a 2D frequency distribution (7.5h). **c** Comparison between methods for calculating population-averaged rates from observational data according to Sun et al., Miller et al. and our own method. Our method depends on a free degradation-associated parameter c_μ that can be expected to assume values around 0.5 or lower (Supplementary Methods 7). The curves show the dependence of the scaled mean absolute deviation (sMAD) relative to the true mean rate as a function of c_μ for $N = 15$ (green), 30 (orange) and 60 (blue). sMADs were calculated using the first 10,000 identified parameter vectors. Notably, for the Rates PAV method in Figure 4 the original model rates were used (i.e. not the above quasi observational data). **d** Total number of molecules per cell and population-averaged production, degradation and growth rates for the illustrating example of Figure 3g ($N = 15$). Growth rates derived from the final (5th) cell cycle phase (equation 2) were quasi identical to the macroscopically derived rates $G = d\log M/dt$ (growth der), with M the cell number. **e**-**f** Analogous results for the illustrating examples of Supplementary Figure 3**g** ($N = 30$) (**e**) and Figure 3k ($N = 60$) (**f**). **g**-**i** Analogous results for the minimum error states (*) (Supplementary Figure 7) for $N = 15$ (**g**), 30 (**h**) and 60 (**i**).

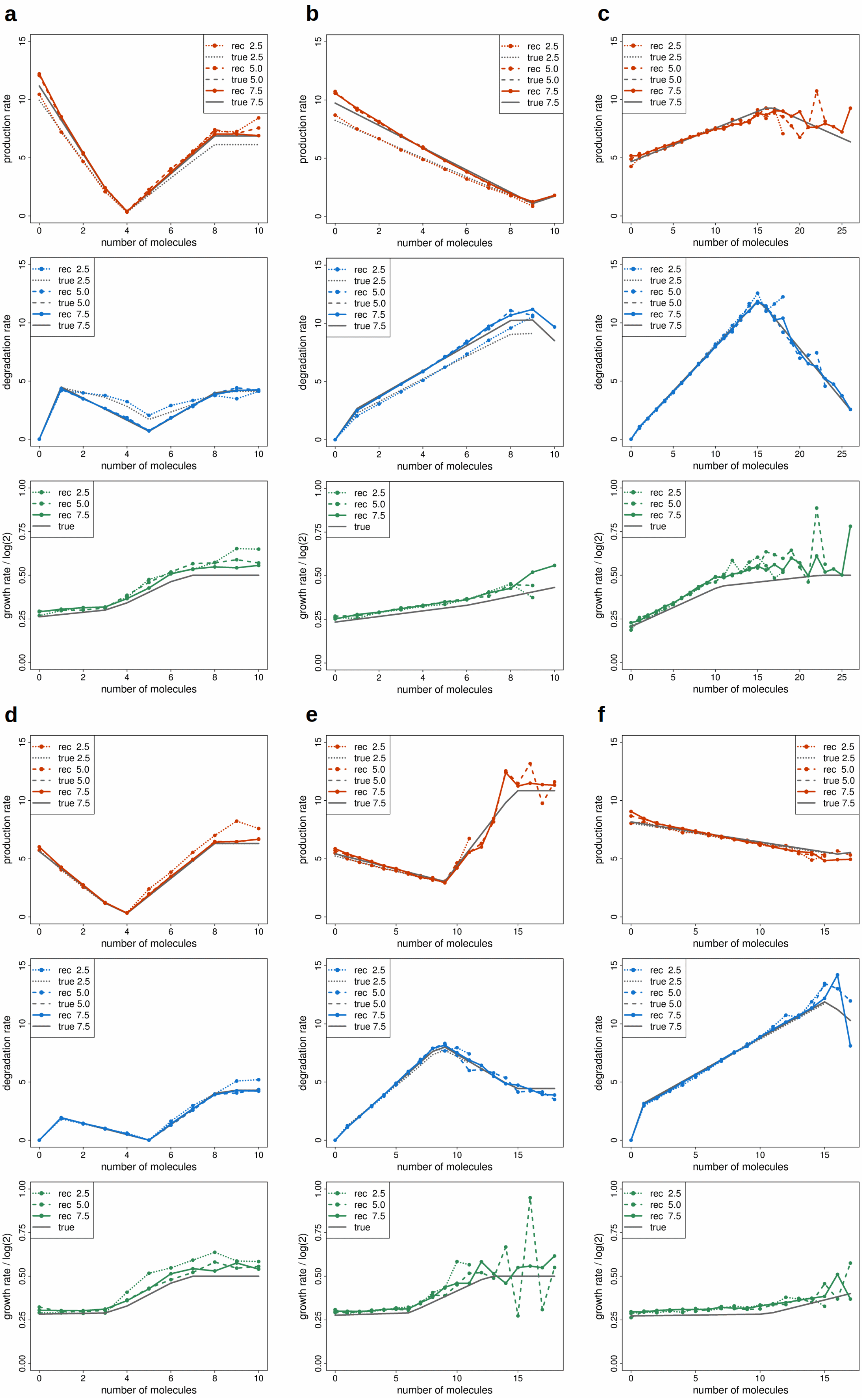
a
b
c
d
e
f
production rate
degradation rate
growth rate / log(2)
number of molecules
rec 2.5
true 2.5
rec 5.0
true 5.0
rec 7.5
true 7.5
true

Supplementary Figure 11. Rate reconstruction from synthetic single cell tracking data. **a** True and reconstructed (rec) production, degradation and growth rates for times 2.5, 5.0 and 7.5h corresponding to the illustrating example of Figure 3**g** for $N = 15$. At 5.0h response induction is mostly complete and results for 5.0 and 7.5h mainly reflect reconstruction variability. **b**-**c** Analogous results for the illustrating examples of Supplementary Figure 3**g** ($N = 30$) (**b**) and Figure 3**k** ($N = 60$) (**c**). **d**-**f** Analogous results for the minimum error states (*) (Supplementary Figure 7) for $N = 15$ (**d**), 30 (**e**) and 60 (**f**). The starting cell number was 50,000. A minimum of 100 observed events per histogram bin were required for evaluation.

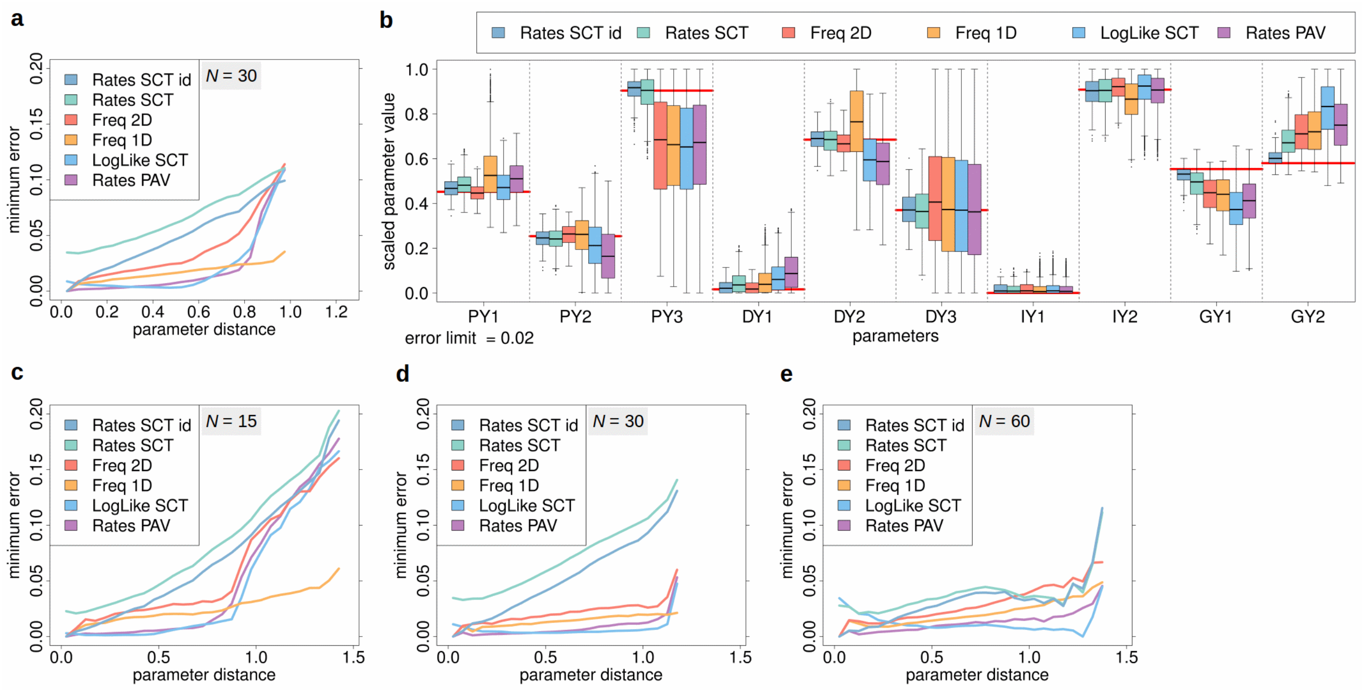

Supplementary Figure 12. a-b Results for $N = 30$ analogous to those for $N = 15$ and $N = 60$ displayed in Figure 5. **c-e** Minimum error versus parameter distance plots analogous to Figure 5**a**,**c** but with parameters PY3 and DY3 included in distance calculations. This decreases the error-distance slopes mostly for those methods that cannot well determine these parameters. See Figure 5 for notation.

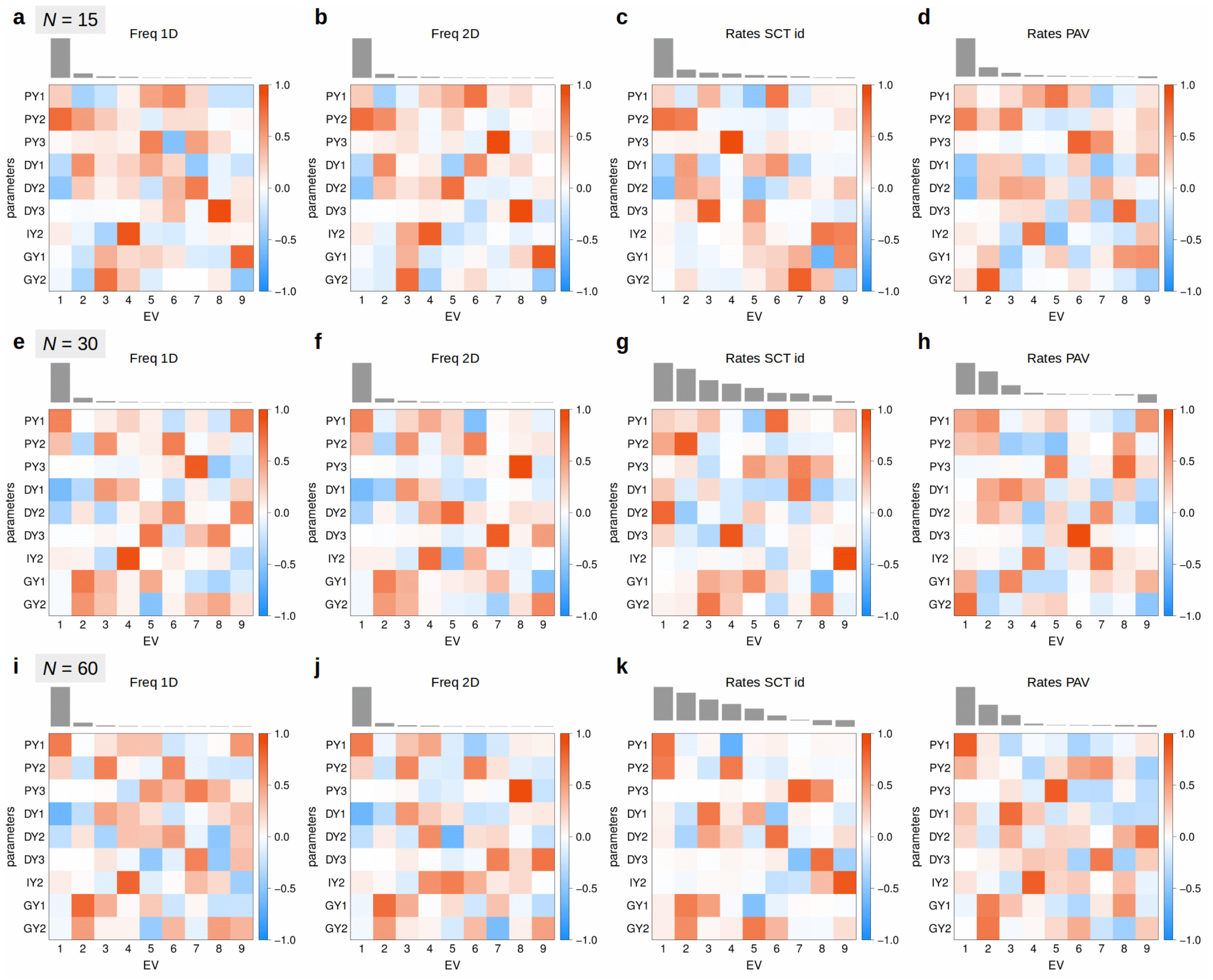

Supplementary Figure 13. Hessian parameter sensitivity analysis. **a** Eigenvalue spectrum (grey barplots on top) and corresponding Eigenvector components (columns) for the Freq 1D method (Figure 3) and $N = 15$. **b**-**d** Analogous results for the methods Freq 2D (**b**), Rates SCT id (**c**) and Rates PAV (**d**). **e**-**l** Analogous results for $N = 30$ (**e**-**h**) and $N = 60$ (**i**-**l**), respectively. The opposite sign of PY1 and PY2 as compared to DY1 and DY2 indicates that the difference between production and degradation is the most important feature for Freq 1D and Freq 2D. Rates SCT id and Rates PAV are expected to show a more reference rate-specific distribution of Eigenvector components. Their similarity to Freq 1D and Freq 2D for $N = 15$ may result from the quasi zero (lower boundary) values of PY2 and DY2 (Figure 5) that imply unidirectional variation. The methods Freq 1D, Freq 2D, Rates SCT id and Rates PAV were selected because they refer to ideal reference data, for which the minimum error is guaranteed to be zero. Projections along Eigenvectors showed that the rare negative Eigenvalues were due to skewed data structures and did not result from saddle points. The y-set-point IY1 was omitted for this reason (skewed and narrow distribution close to zero; Figure 5).

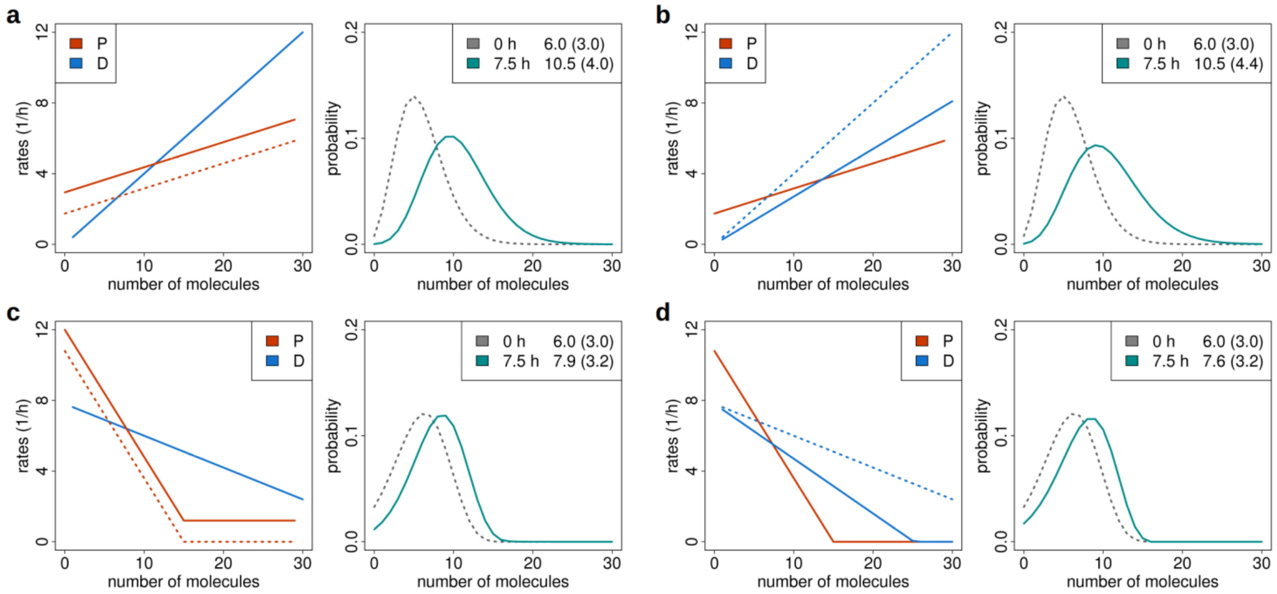

Supplementary Figure 14. Response of additional regulation types to identical treatment. **a**-**d** Constant increase in production rate (**a**,**c**) or decrease in molecular degradation rate (**b**,**d**) applied to the ground state of two scenarios, in which both P and D either increase (**a**,**b**) or decrease (**c**,**d**) with molecule number. For both models, the ground state standard deviation (3.0) is larger than in Figure 6 (2.4,2.8) and its positive shift is larger in **b** (1.4) as compared to Figure 6**b** (0.9). Otherwise, responses are similar to those in Figure 6. For notation see Figure 6.
